# Association between cholecystectomy/gallbladder pathology and colorectal polyps: a systematic review and meta-analysis

**DOI:** 10.3389/fonc.2025.1724606

**Published:** 2026-01-14

**Authors:** Shirui Li, Yunfan Deng, Sheng Dai, Qi Fan, Dehai Xiong, Xiuyang Li

**Affiliations:** 1 Department of Big Data in Health Science, and Center for Clinical Big Data and Statistics, The Second Affiliated Hospital, College of Medicine, Zhejiang University, Hangzhou, Zhejiang, China; 2 Zhejiang Key Laboratory of Intelligent Preventive Medicine, Zhejiang University School of Medicine, Hangzhou, China; 3 Department of General Surgery, Affiliated Sir Run Run Shaw Hospital, Zhejiang University School of Medicine, Hangzhou, China; 4 Department of General Surgery, Three Gorges Hospital, Chongqing University, Chongqing, China

**Keywords:** colorectal (colon) cancer, colorectal polyps, gallbladder disease, gallbladder polyp, gallstone, meta-analysis

## Abstract

**Background:**

Gallbladder-related pathologies may influence colorectal carcinogenesis, yet systematic evaluation of their associations with precursor lesions remains limited. This study addresses critical knowledge gaps by investigating the dual-axis relationship between cholecystectomy/gallbladder pathologies and colorectal polyp risk while elucidating geographical and biological effect modifiers.

**Methods:**

In accordance with the MOOSE/Cochrane guidelines, 27 observational studies were analysed through PROSPERO (CRD420251012876). Comprehensive meta-analysis was performed to assess the effects of cholecystectomy across polyp subtypes and gallbladder pathology (stones/polyps) associations. Heterogeneity was quantified via *I²* statistics, and pooled odds ratios (ORs) with 95% confidence intervals (CIs) were calculated via the DerSimonian–Laird random effects model to account for between-study variance. Subgroup analysis stratified by geography, pathology type, and adjustment model was performed with *χ²* tests for subgroup differences. Sensitivity analysis and publication bias were assessed through leave-one-out methods, funnel plots and Egger’s test.

**Results:**

Cholecystectomy was associated with a 39% increased risk (OR = 1.39, 95% CI: 1.21–1.59), with East Asian populations exhibiting a nearly doubled risk compared with North American populations (OR=1.95(95%CI:1.44-2.63) vs OR=1.16(95%CI:1.00-1.34). Compared with unclassified polyps, adenomas were more strongly associated (OR = 1.37, 95% CI: 1.16–1.62). Medium-sized studies (OR = 1.69, 95% CI: 1.36–2.11) and those adjusted for health factors (OR = 1.34, 95% CI: 1.12–1.61) yielded higher estimates, whereas dietary adjustment nullified significance. Gallbladder pathology (stones/polyps) conferred a 27% higher risk overall, with gallbladder polyps showing greater risk (OR = 1.30, 95% CI: 1.17–1.38) than stones (OR = 1.20, 95% CI: 1.08–1.32). Older populations (≥50 years) had stronger associations OR=1.41(95%CI:1.20-1.66) vs OR=1.20(95%CI:1.10-1.30). Adjustment for smoking, alcohol, and BMI strengthened the estimates (OR = 1.43, 95% CI: 1.22–1.68). Sensitivity analysis supported the robustness of the primary findings, particularly for cholecystectomy.

**Conclusions:**

This comprehensive analysis establishes gallbladder status as an independent risk modulator for early colorectal lesions, with cholecystectomy demonstrating the highest risk magnitude. These findings advocate personalized surveillance strategies that integrate gallbladder history, particularly high-risk demographics. The mechanistic synergies between bile acid dysmetabolism and gut microbiota dysbiosis warrant further exploration as preventive targets.

**Systematic review registration:**

https://www.crd.york.ac.uk/prospero/, identifier CRD420251012876.

## 1 Introduction

Colorectal cancer (CRC), the third most prevalent malignancy worldwide, accounted for approximately 1.92 million new cases and 900,000 deaths in 2022, with Asia bearing the highest regional burden (1). Sporadic CRC predominantly arises from the adenoma–carcinoma sequence, wherein colorectal polyps (CRPs), particularly adenomatous polyps (APs) and serrated polyps (SPs), serve as critical precursor lesions (2–4). While conventional risk factors encompassing *APC* gene mutations, aging, and dietary patterns (particularly red meat consumption) remain well characterized (5, 6), emerging evidence implicates gallbladder-related pathologies as potential modulators of colorectal carcinogenesis. The proposed biological link primarily involves perturbations in bile acid enterohepatic circulation and gut microbial homeostasis, which may collectively promote a protumorigenic microenvironment in the colorectum (7).

Previous meta-analyses on CRC and gallbladder factors have yielded inconsistent results. For cholecystectomy (CC), some studies reported a modestly elevated CRC risk (RR = 1.22) (8) or site-specific increases (e.g., sigmoid colon, RR = 1.42) (9), whereas others found no significant association with precancerous adenomas (RR = 1.17, 95% CI: 0.93–1.48) (10), suggesting stage-specific effects. Similarly, gallstone disease suffers from endpoint bias; Polychronidis et al. reported moderately increased CRC risk (RR = 1.15) but did not analyze further polyp formation (11). This study design flaw is even more prominent for gallbladder polyps, as CRC development usually requires a long latency period, and it is difficult to capture the driving effect of gallbladder mucosal hyperplasia on early colorectal mucosal lesions using CRC as an endpoint. However, some screening cohorts have reported a 40% increased risk of colorectal adenoma in patients with gallbladder polyps (12), supporting a potential role in early carcinogenesis.

This controversy has highlighted several critical limitations in existing research. First, exposure classification is heterogeneous. For example, gallstones and gallbladder polyps are often grouped under the broad category of “gallbladder disease,” overlooking their distinct pathological mechanisms and, consequently, their potentially different roles in carcinogenesis. Specifically, gallstones primarily reflect a disorder of bile chemistry and gallbladder motility, whereas gallbladder polyps represent mucosal growths frequently driven by localized inflammation. This pathophysiological distinction suggests that they may influence colorectal mucosa through related yet distinct pathways (e.g., chronic bile acid exposure versus systemic inflammatory mediators), justifying their separate evaluation in our analysis (13). Similarly, cholecystectomy is frequently conflated with the underlying pathology, confounding risk estimation (14, 15). Second, the classification of endpoint events is overly broad. While most meta-analyses focus on CRC or colorectal neoplasms (CRNs), systematic investigations into CRPs, a precursor lesion, remain insufficient. This narrow focus limits the understanding of early-stage disease progression. Third, the reporting of key effect modifiers is notably inadequate. Insufficient original data on variables such as the time interval since cholecystectomy, gallstone size, or polyp anatomic location. This lack of granularity restricts the analysis of dose–response relationships and time-dependent risk, hindering the identification of potential causal pathways and effect modifiers.

To overcome these limitations, we propose a two-pronged approach to simultaneously examine the effects of cholecystectomy and gallbladder disorders on CRPs risk. This study specifically aims to: 1) examine the relationship between cholecystectomy and the risk of CRPs across histological subtypes (adenoma, hyperplasia, and serrated); and 2) investigate the relationship between gallbladder disorders and CRPs, independently assess the effects of gallstones and polyps on polyp formation, and differentiation of the independent effects of chronic gallbladder inflammation and anatomical loss. The aim is to avoid misjudgment of effects due to confusion of anatomical changes with the pathological status of the organ and provide clearer clinical guidance. Methodologically, we combined meta-analysis—to quantify effect sizes where data were sufficient—with systematic review for a qualitative synthesis of factors with limited studies. This framework provides a novel perspective on the gallbladder–gut axis and a scientific basis for risk-stratified clinical surveillance.

## 2 Methods

The Meta-analysis of Observational Studies in Epidemiology guidelines and Cochrane’s Handbook were followed in this study. The study protocol was prospectively registered (CRD420251012876) prior to data extraction, ensuring transparency and reducing selection bias.

### 2.1 Data sources and search strategy

A systematic search was conducted in the PubMed, Embase, Cochrane Library, Web of Science, and Chinese databases (CNKI, Wanfang, SinoMed) from inception to February 2025, with language restrictions to English and Chinese. We acknowledge that this may introduce language bias, but it was necessary due to practical constraints in translation and resources. The search strategy focused on two themes: (1) gallbladder diseases and surgical interventions (e.g., “gallstones,” “gallbladder polyps,” “cholecystectomy”) and (2) colorectal polyps (e.g., “adenomatous polyps,” “serrated lesions”). We combined MeSH terms (“Gallbladder Diseases,” “Gallstone,” “Colonic Polyps,” “Adenoma”) with free-text keywords via Boolean operators (AND/OR). We did not systematically search grey literature (e.g., conference abstracts, preprints) to ensure the inclusion of only peer-reviewed, full-text articles with complete methodologies and data necessary for rigorous quality assessment and meta-analysis. This approach enhances the reliability of our pooled estimates but may have missed some very recent or unpublished findings. The full search strategies are provided in **Supplementary Material 1**.

### 2.2 Study selection

**Figure 1:**
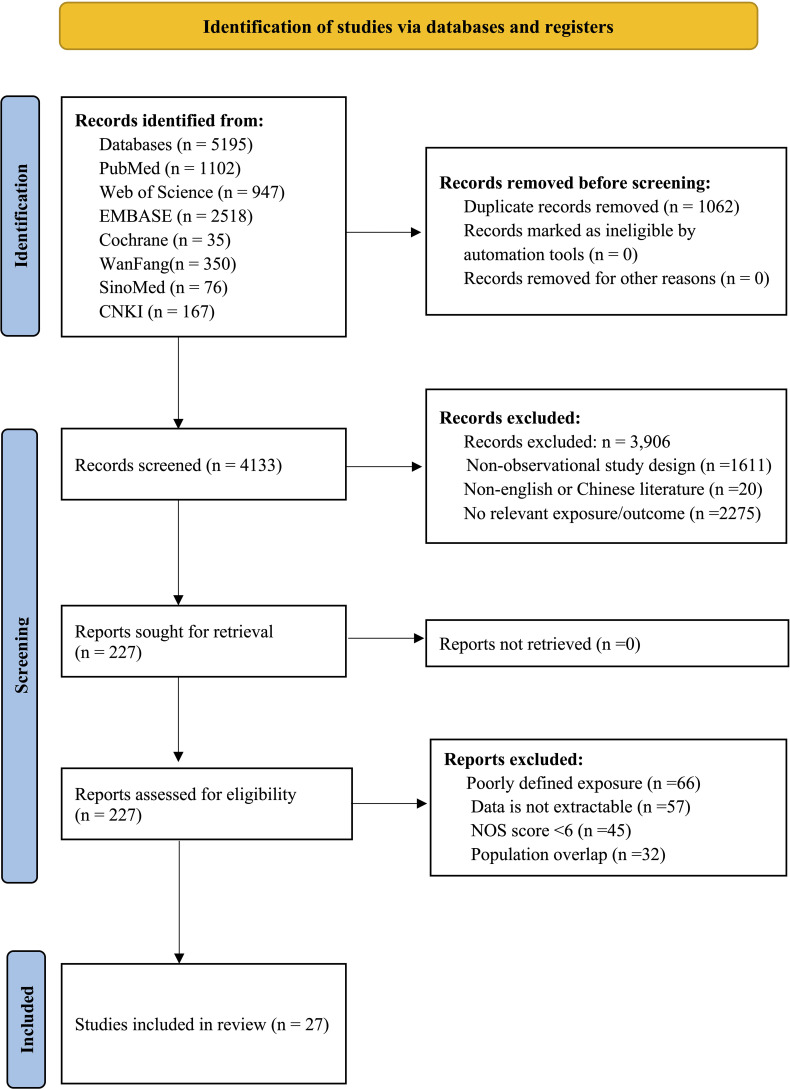
PRISMA flow diagram.

Records were imported into EndNote Web v.20 for deduplication. Two independent reviewers (Li and Deng) screened titles/abstracts via Rayyan, a systematic review management platform, with full-text review for ambiguous cases. Discrepancies were resolved by a third reviewer (Lee). Eligible studies were observational in design (cohort, case–control, or cross-sectional) and investigated associations between specifically defined gallbladder disorders (gallstone, gallbladder polyps, or cholecystectomy) and incident colorectal polyps. Studies merely describing “gallbladder disease” without subtype specification were excluded. Exposures required objective validation: gallstones by imaging/surgical confirmation, gallbladder polyps through ultrasound/CT/MRI/endoscopic ultrasound, or cholecystectomy via surgical records. However, the variability in diagnostic accuracy across these modalities, particularly for polyp detection, represents a potential source of exposure misclassification and between-study heterogeneity. The outcomes included histologically confirmed non-malignant colorectal polyps (adenomas, hyperplastic polyps, serrated polyps, unclassified polyps). We excluded non-observational studies, non-English/Chinese publications, duplicate reports, and studies focused solely on invasive carcinomas. Articles lacking validated exposure measures, insufficient outcome data for effect size calculation, or ambiguous gallbladder disease classification were also excluded. More detailed inclusion and exclusion criteria are presented in **Supplementary Material 2**. The selection process followed PRISMA guidelines (**Figure 1**).

### 2.3 Data extraction

Two independent reviewers used a standardized spreadsheet to extract data: study identifiers, histopathological classification of colorectal polyps, gallbladder disease status, epidemiological study designs, and covariate adjustment. Geographical distribution was documented by continent and country of origin. The demographic parameters included the total sample size, age distribution metrics, and sex composition. The temporal characteristics included the recruitment timeframe, study duration, and publication chronology. For studies reporting multiple polyp subtypes, subtype-specific effect estimates were preserved in discrete data fields. The quantitative measures consisted of risk estimates with 95% CIs supplemented by logarithmic transformations for meta-analytic synthesis. For subgroup analysis, subgroups with fewer than three studies were excluded, and those missing values for a variable (such as the mean age or sex ratio) were excluded from the subgroup analysis and meta-regression.

### 2.4 Evaluating the quality of the studies

The quality of the included observational studies was assessed via the Newcastle–Ottawa Scale (NOS), a validated tool specifically designed for evaluating the methodological quality of nonrandomized studies, including cohort and case–control studies. Each study was awarded a maximum of 9 stars, with higher scores indicating higher methodological quality. The NOS evaluates studies across three domains:

1. Selection (4 stars): Assesses the representative of the exposed cohort, selection of the nonexposed cohort, ascertainment of exposure, and demonstration that the outcome of interest was not present at the start of the study.
2. Comparability (2 stars): Evaluates the control for confounding factors, particularly whether the study is adjusted for age, sex, and other relevant variables.
3. Outcome (3 stars): Assesses the method of outcome assessment, the adequacy of follow-up, and whether the follow-up was long enough for outcomes to occur.

The interviewer consistency kappa was 0.85, and disagreements were resolved through consultation.

### 2.5 Statistical analysis methods

Heterogeneity was evaluated via I² statistics and Cochran’s Q test (significance threshold: *I²*≥50% or Q test *p* < 0.10), guiding selection between random effects and fixed effects models. Primary meta-analyses were conducted in *Review Manager 5.4* (Cochrane Collaboration) via the DerSimonian–Laird random effects model to account for between-study variance. The sensitivity analysis included leave-one-out analysis, a quality-based analysis in which studies with low NOS scores (<6) were excluded, and restricted maximum likelihood (REML) estimation and Hartung-Knapp adjustments were implemented in *R 4.3.0* via the *metafor* package (v3.8-1). Publication bias was assessed via funnel plots, Egger’s regression test, and Begg’s test. The trim-and-fill method was used to estimate and adjust for potential publication bias. Univariable meta-regression was performed to explore associations between study-level covariates and the effect size. The proportion of variance explained (R²) was calculated for each covariate. A detailed classification of all subgroup analyses and statistical methods is provided in **Supplementary Material 3**. All the statistical tests were two-tailed (*α* = 0.05).

## 3 Result

### 3.1 Cholecystectomy and colorectal polyps

#### 3.1.1 Study selection and characteristics

This meta-analysis synthesized evidence from 21 observational studies (25 effect estimates) involving 405,527 participants across three continents, with publication dates spanning 1984-2024. Geographically, 10 studies originated from China, 8 studies from North America, and 3 studies from other regions, including Japan and Australia. Methodologically, case–control designs predominated (n=10), followed by cohort studies (n=8) and cross-sectional investigations (n=3). Sample sizes demonstrated substantial heterogeneity, ranging from 144 participants in an Australian case–control study to 154,224 subjects in a U.S. cohort. Quality assessment via the Newcastle–Ottawa Scale revealed moderate methodological rigor (median score=7), with post-2010 studies showing improved quality scores (53.8% with NOS≥8 vs. 33% in earlier research). The complete study characteristics are tabulated in **Table 1**.

**Table 1:** Characteristics of studies included in cholecystectomy-colorectal polyp association.

Study (Year)	Pathology type	Study design	Adjustment*	Region	Sample size	Recruitment period	NOS score	Citation
Yamaji 2008	Adenoma	Cross-sectional	D+H	Japan	5,719	1991-2003	9	(16)
Guo 2023	Adenoma	Case-Control	H+N	China	1,149	2016-2020	7	(17)
Zhang 2021	Unclassified	Case-Control	H	China	242	2018-2020	6	(18)
Chen 2017	Adenoma	Cross-sectional	D+	China	1,125	2003-2012	7	(19)
Dong 2024	Adenoma	Case-Control	H+N	China	676	2016-2023	9	(20)
Wang 2021	Adenoma	Case-Control	D+H	China	743	2014-2018	8	(21)
Sandler 1988	Adenoma	Case-Control	D+N	USA	1,407	1983-1985	8	(22)
Schernhammer 2003	Adenoma	Cohort	D+H+N	USA	85,184	1982-1998	9	(23)
Neugut 1988	Adenoma	Case-Control	D+	USA	400	1983-1985	6	(24)
Siddiqui 2009	Adenoma	Case-Control	D+	USA	1,234	2000-2007	8	(25)
Vinikoor 2008	Adenoma	Case-Control	D+H	USA	1,437	1998-2002	8	(26)
Kahn 1988	Unclassified	Cohort	D+H	USA	154,224	1982-1992	8	(15)
Neugut 1991	Adenoma	Case-Control	D+	USA	2,750	1986-1988	9	(27)
Wang 2023	Serrated poly	Cohort	D+H	China	761	2019-2022	9	(28)
Polychronidis 2021	Adenoma/Serrated poly	Cohort	D+H+N	USA	133721	1991-2012	7	(29)
Xu 2011	Adenoma	Cohort	D+	China	11,863	2008-2011	7	(30)
Llamas 1986	Adenoma	Case-Control	D+	Australia	144	1980-1982	6	(31)
Mannes 1984	Adenoma	Cohort	Unreported	Germany	761	1976-1982	9	(32)
Zhuang 2011	Adenoma	Cohort	Unreported	China	503	2009-2010	9	(33)
Wang 2017	Adenoma	Cross-sectional	Unreported	China	702	2013-2016	7	(34)
Luo 2014	Adenoma	Cohort	Unreported	China	981	2007-2013	7	(35)

*Adjustment models: D, Demographic factors (age, sex); H, Health factors (smoking, alcohol, BMI); N, Nutritional factors.

#### 3.1.2 Cholecystectomy and colorectal polyp risk

**Figure 2:**
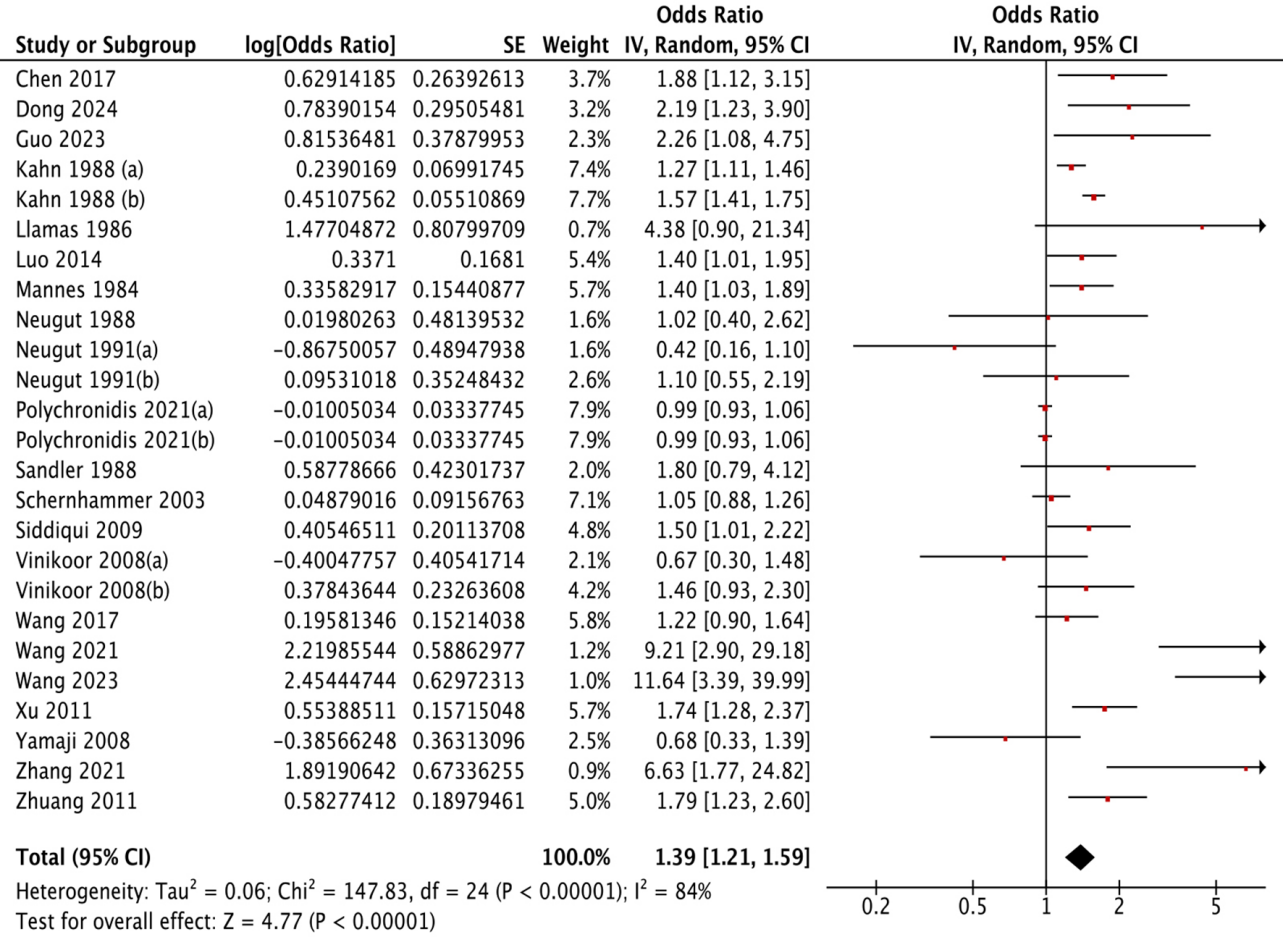
Association of gallbladder conditions with colorectal polyps.

Cholecystectomy was associated with a 39% greater risk of colorectal polyps (OR = 1.39, 95% CI: 1.21–1.59; I² = 84%, *P* < 0.001) (**Figure 2**). The 95% prediction interval, which estimates the range within which the true effect size would fall in 95% of future similar settings, was 0.84 to 2.29. This wide interval reflects the substantial heterogeneity observed across studies.

**Figure 3:**
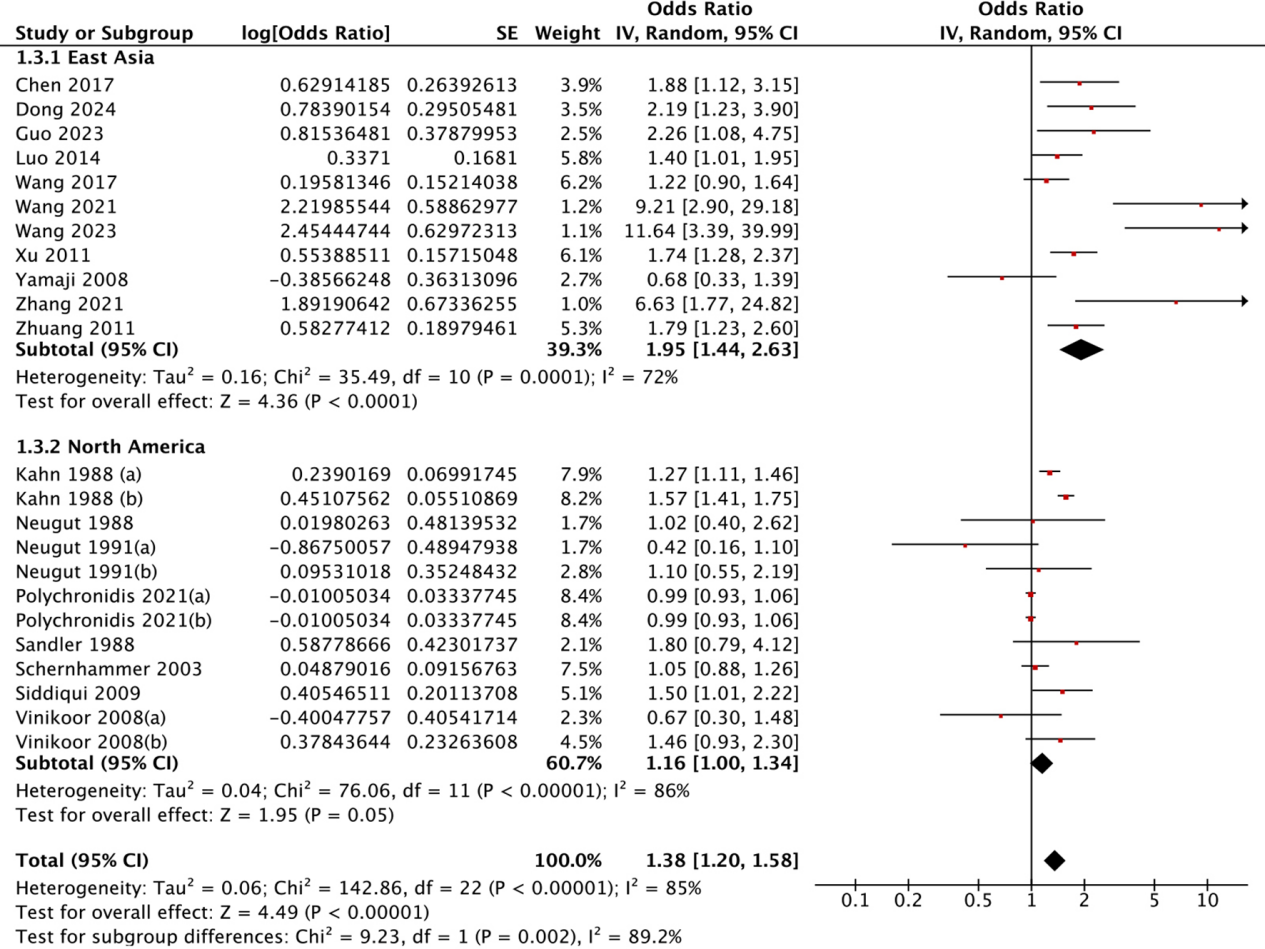
Subgroup analysis by geographic region: association between cholecystectomy and colorectal polyp risk.

**Figure 4:**
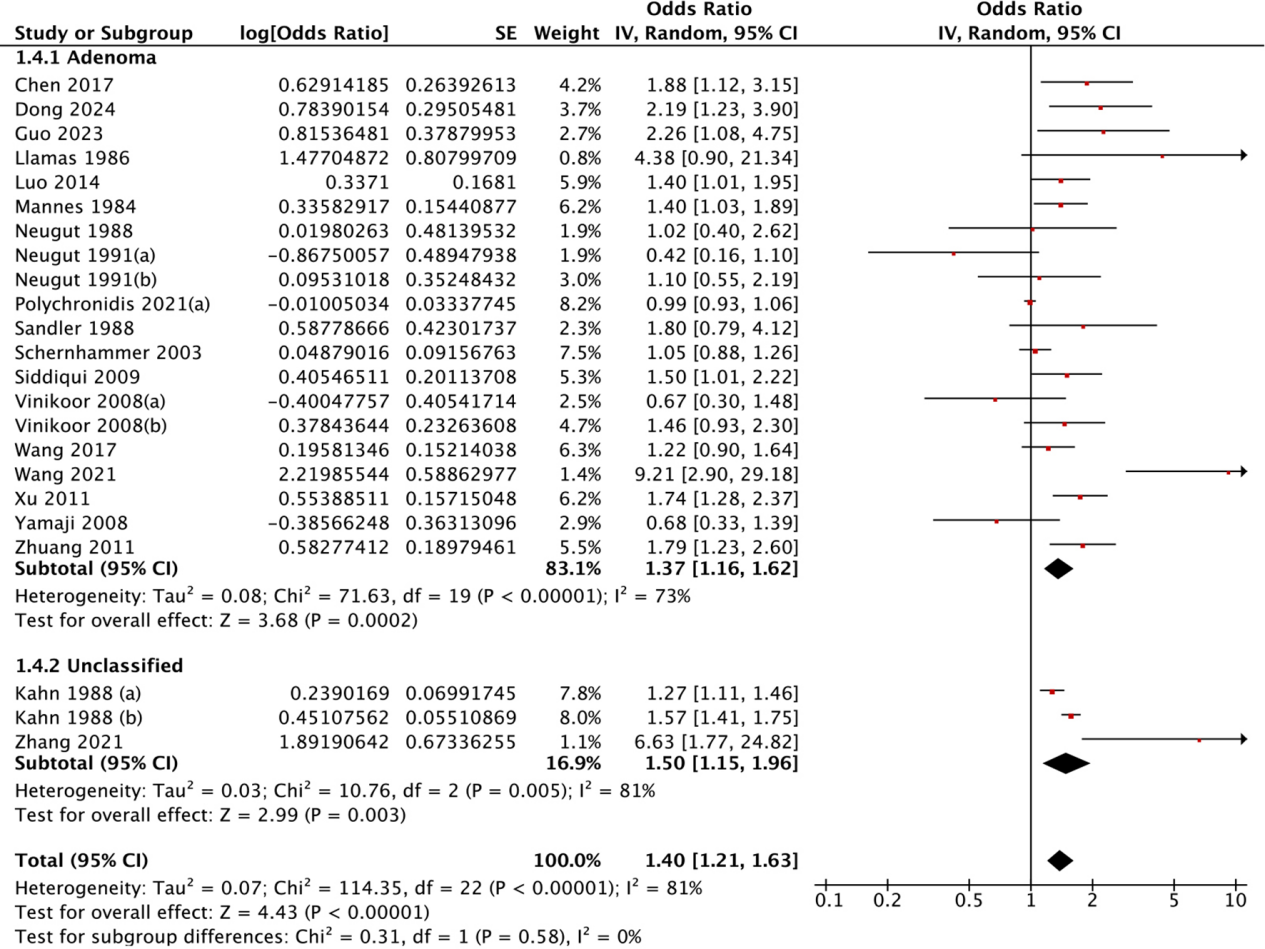
Subgroup analysis by polyp pathology: association between cholecystectomy and colorectal polyp risk.

**Figure 5:**
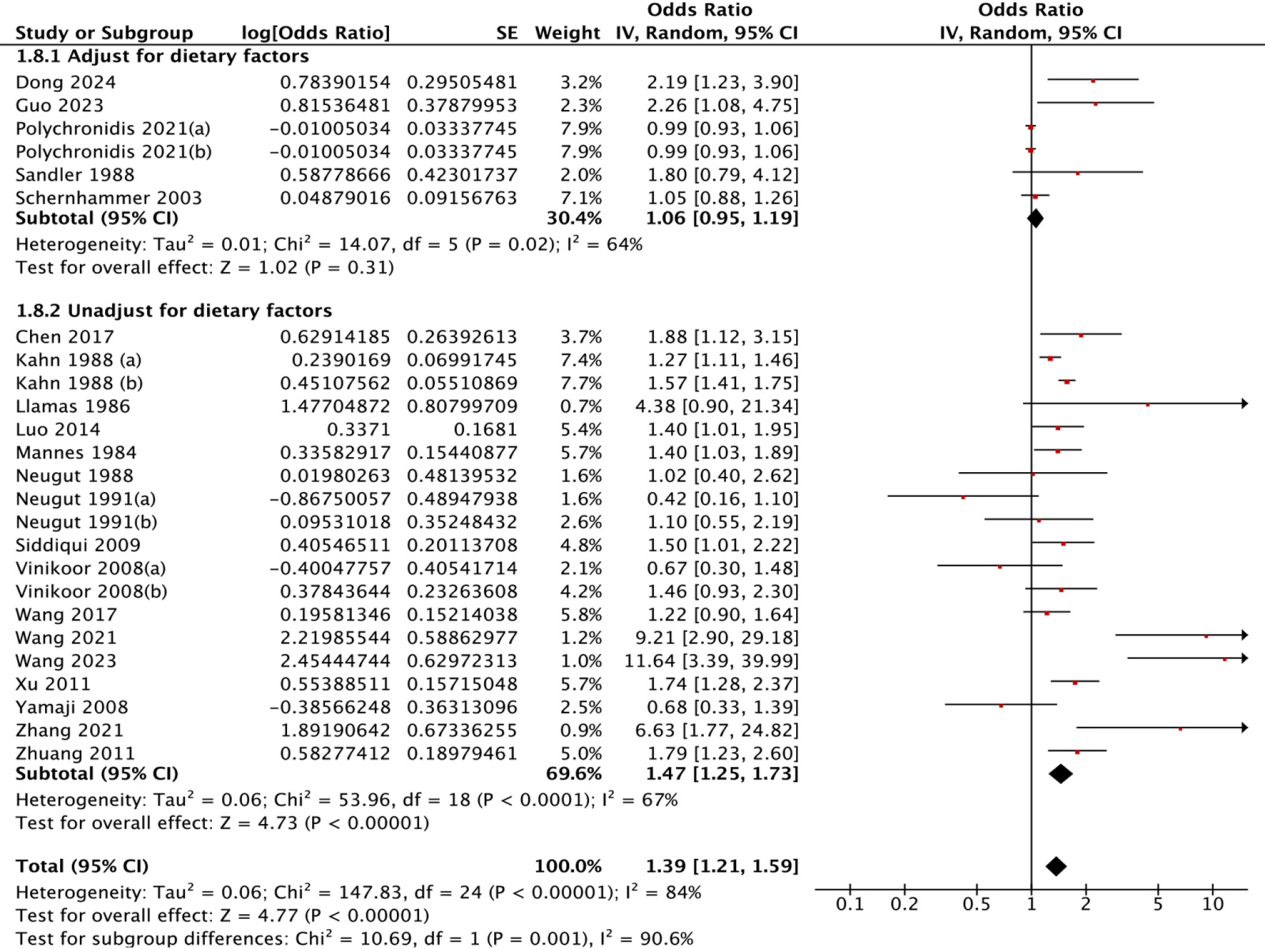
Subgroup analysis by dietary adjustments: association between cholecystectomy and colorectal polyp risk.

Geographical disparities were evident. East Asian populations demonstrated significantly greater risks (OR = 1.95, 95% CI: 1.44-2.63) than North American cohorts did (OR = 1.16, 95% CI: 1.00-.34). This geographical disparity was statistically confirmed through heterogeneity testing (Chi²=9.23, *P* = 0.002) (**Figure 3**). In terms of polyp pathology, adenoma-specific studies demonstrated a pooled OR of 1.37 (95% CI: 1.16-1.62), whereas studies of unclassified types revealed significant associations (OR = 1.50, 95% CI: 1.15-1.96) (**Figure 4**). Furthermore, the strength of association varied with the adjustment for different confounders. Models adjusting for health factors (e.g., smoking, BMI) yielded an OR of 1.34 (95% CI: 1.12-1.61), while further adjustment for dietary factors attenuated the effect to non-significance (OR = 1.06, 95% CI: 0.95-1.19) (**Figure 5**). Analyses of other methodological factors, including study sample size, are presented in **Supplementary Figure S1** and **Supplementary Table S1**.

#### 3.1.3 Sensitivity analysis and publication bias

**Figure 6:**
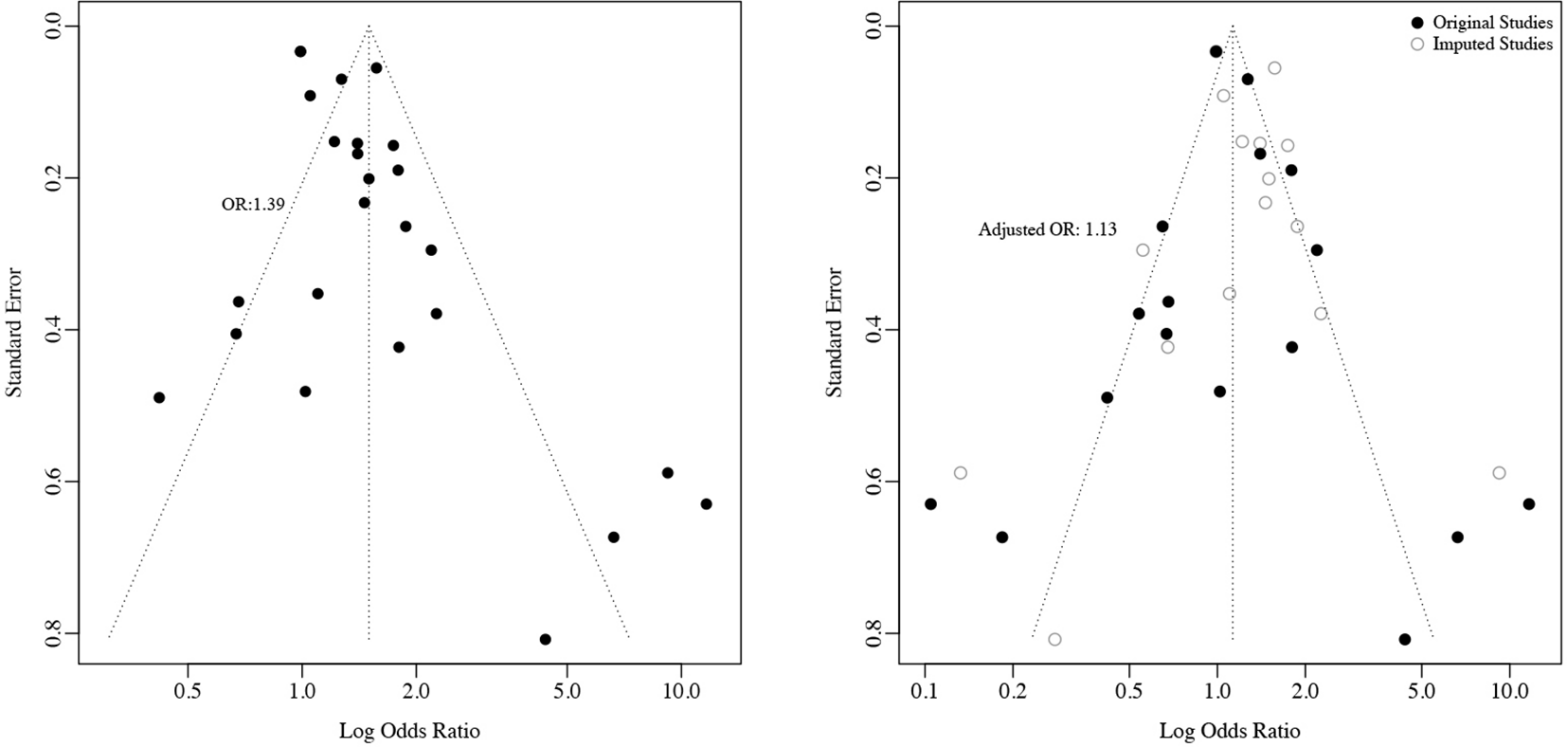
Publication Bias assessment and trim-and-fill adjustment: **(a)** Funnel Plot **(b)** Trim and Fill adjusted effect Size Distribution.

Leave-one-out iterations produced consistent effect estimates (OR range: 1.41-1.54; all *P* < 0.05), with no single study disproportionately influencing the pooled estimate (**Table 2**). Egger’s test suggested potential small-study effects (*P* = 0.005), whereas Begg’s test revealed no significant publication bias (*P* = 0.469). Trim-and-fill adjustment imputing 8 hypothetical studies yielded an attenuated but still significant effect size (OR = 1.13, 95% CI: 1.01-1.27), indicating that while the observed effect might be slightly overestimated, the positive association remains statistically robust (**Figure 6**). The cumulative meta-analysis demonstrated relative stabilization of effect estimates over time (OR = 1.50, 95% CI: 1.15-1.95), despite early fluctuations caused by imprecise estimates in pioneering studies (**Supplementary Table S2**).

**Table 2:** Leave-one-out sensitivity analysis results.

Study	OR	95% CI_lower	95% CI_upper
Omitting Yamaji 2008	1.54	1.18	2.01
Omitting Guo 2023	1.48	1.12	1.95
Omitting Zhang 2021	1.44	1.12	1.85
Omitting Chen 2017	1.49	1.13	1.97
Omitting Dong 2024	1.48	1.12	1.95
Omitting Wang 2021	1.41	1.12	1.78
Omitting Sandler 1988	1.50	1.13	1.98
Omitting Schernhammer 2003	1.53	1.16	2.02
Omitting Neugut 1988	1.52	1.16	2.01
Omitting Siddiqui 2009	1.51	1.14	1.99
Omitting Vinikoor 2008 (a)	1.54	1.18	2.01
Omitting Vinikoor 2008 (b)	1.51	1.14	2.00
Omitting Kahn 1988 (a)	1.52	1.15	2.01
Omitting Kahn 1988 (b)	1.50	1.13	1.99
Omitting Neugut 1991 (a)	1.54	1.20	1.98
Omitting Neugut 1991 (b)	1.52	1.15	2.01
Omitting Wang 2023	1.41	1.13	1.76
Omitting Polychronidis 2021 (a)	1.54	1.17	2.03
Omitting Polychronidis 2021 (b)	1.54	1.17	2.03
Omitting Xu 2011	1.49	1.13	1.98
Omitting Llamas 1986	1.47	1.13	1.92
Omitting Mannes 1984	1.51	1.14	2.00
Omitting Zhuang 2011	1.49	1.13	1.97
Omitting Wang 2017	1.52	1.15	2.01
Omitting Luo 2014	1.51	1.14	2.00
Pooled estimate	1.50	1.15	1.95

### 3.2 Gallbladder pathology and colorectal polyp

#### 3.2.1 Study selection and characteristics

Ten studies (pooled sample: 133,721 participants) investigated associations between gallbladder pathology (stones/polyps) and colorectal polyps. Geographical distribution revealed strong Asian representation, with one U.S. cohort broadening demographic diversity. Methodologies included cross-sectional, case–control and cohort designs, spanning diverse age groups (<50 years: 5 studies; ≥50 years: 5 studies), with sample sizes spanning two orders of magnitude (103-133,721 participants). Methodological quality and recruitment periods showed substantial variability (**Supplementary Table S3**).

#### 3.2.2 Gallbladder pathology and colorectal polyp risk

The pooled analysis demonstrated a modest association between gallbladder pathology and colorectal polyps (OR = 1.27, 95% CI: 1.17–1.38; *I*² = 64%, *P* < 0.001). The 95% prediction interval for this association was 0.95 to 1.70.

**Figure 7:**
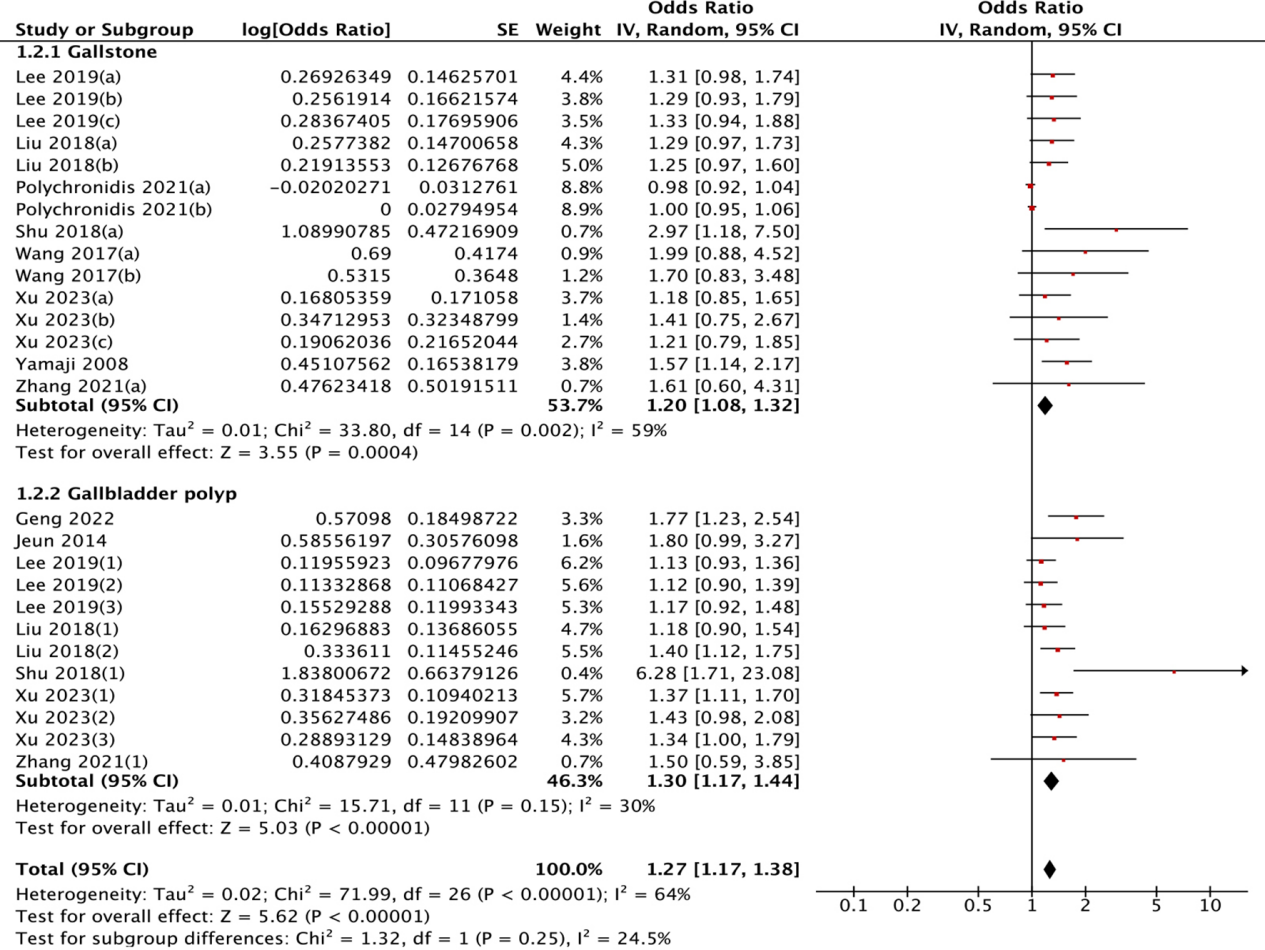
Differential impact of gallbladder pathology subtypes on colorectal polyp risk.

**Figure 8:**
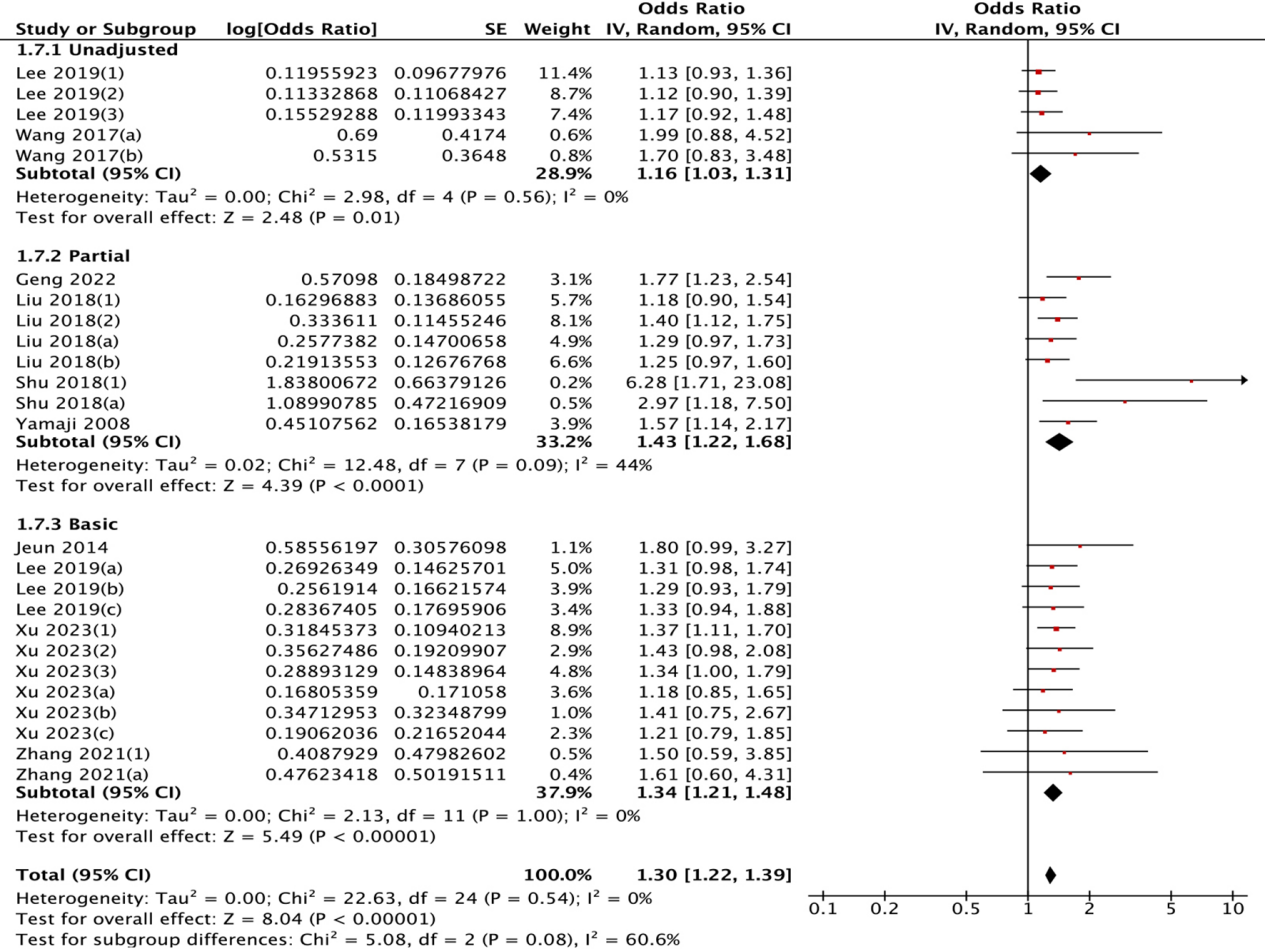
Subgroup analysis of association between cholecystectomy and colorectal polyp risk. **(a)** by gender; **(b)** by sample size; **(c)** by adjustment for basic factors (sex/age); **(d)** by adjustment for healthy factors (alcohol/ smoking/ BMI).

Stratification by pathology type revealed differential risks: gallstones (OR = 1.20, 95% CI: 1.08–1.32) versus gallbladder polyps (OR = 1.30, 95% CI: 1.17–1.44) (**Figure 7**). Age-stratified analysis demonstrated stronger associations in older populations (≥50 years: OR = 1.41, 95% CI: 1.20-1.66) than in younger subgroups (OR = 1.20, 95% CI: 1.10-1.30), although this difference did not reach formal statistical significance (*P* = 0.07) (**Supplementary Figure S2**). A clear pattern emerged regarding confounding control: more comprehensive adjustment models yielded stronger associations. The risk estimate increased from OR = 1.16 (unadjusted) to OR = 1.34 (adjusted for age/sex), and was strongest (OR = 1.43, 95% CI: 1.22–1.68) in models that additionally accounted for smoking, alcohol, and BMI (**Figure 8**). Meta-regression identified that higher study quality (NOS score) was a significant inverse predictor of the risk estimate (β = -0.19 per point, P < 0.001), suggesting that methodological rigor may attenuate the observed effect. Full meta-regression results are available in **Supplementary Table S4**. Analyses of other methodological factors, including study type, are presented in **Supplementary Figure S2**.

#### 3.2.3 Sensitivity analysis and publication bias

Leave-one-out sensitivity analysis confirmed robustness (OR = 1.24-1.28; all *P* < 0.001). However, trim-and-fill adjustment imputing 14 studies attenuated the effects to no significance (OR = 1.09, 95% CI: 0.96–1.23), revealing potential publication bias and suggesting that the observed association for gallbladder pathology requires more cautious interpretation and further validation (**Supplementary Table S5**).

### 3.3 Certainty of the evidence

The certainty of the evidence was assessed via the GRADE (Grading of Recommendations, Assessment, Development, and Evaluations) framework. The certainty was further graded on the basis of the risk of bias (assessed via the Newcastle–Ottawa Scale), inconsistency (evaluated via I² statistics), indirectness, imprecision (wide confidence intervals), and publication bias (assessed via funnel plots, Egger’s test, and Begg’s test). The final certainty of the evidence for each outcome is presented in the summary table (**Supplementary Table S6**).

## 4 Discussion

### 4.1 Main findings and risk gradient

Our study revealed a significant association between cholecystectomy and colorectal polyp risk (OR = 1.39, 95% CI: 1.21-1.59), with a greater effect magnitude than that reported in previous meta-analyses on CRC outcomes. Notably, although earlier meta-analyses failed to identify significant CC-CRPs associations (RR = 1.17, 95% CI: 0.93-1.48), our findings revealed greater effect sizes for cholecystectomy on adenomas (OR = 1.37, 95% CI: 1.16-1.62) than for unclassified polyps, suggesting stage-specific gallbladder involvement in the adenoma–carcinoma sequence. However, given that the annual malignant transformation rate of adenomas ranges from merely 0.25%-6%, with variation by age, sex, and surveillance duration (36), cautious interpretation remains warranted regarding the CRC implications of gallbladder–polyp associations. Through our two-pronged analysis and subgroup stratification, we identified a risk gradient (CC OR = 1.39 > gallbladder polyp OR = 1.30 > gallstone OR = 1.20), indicating that anatomical irreversibility and distinct inflammatory patterns of gallbladder pathology potentially constitute key drivers of risk stratification.

### 4.2 Biological plausibility and proposed mechanisms

Post-cholecystectomy bile acid enterohepatic cycling acceleration significantly elevates secondary bile acid proportions (e.g., DCA and LCA) through continuous biliary flow into the intestine (37). These hydrophobic bile acids directly damage enterocyte membranes while inducing reactive ROS/RNS generation, triggering oxidative–nitrosative stress that induces DNA damage and promotes proliferative signaling via the Wnt/β-catenin pathway, thereby driving adenoma formation (38–40). This is compounded by concomitant gut microbiota dysbiosis, characterized by a reduction in beneficial, short-chain fatty acid-producing bacteria and an expansion of pathobionts, which impairs intestinal barrier integrity, LPS translocation and amplifies systemic low-grade inflammation, insulin resistance, and lipid metabolism disorders (41–43). A vicious cycle emerges through bile acid–microbiota crosstalk: increased bacterial BSH activity accelerates primary bile acid deconjugation, further increasing DCA production, enriching pathobionts to exacerbate the imbalance in the mucosal microenvironment (44, 45).

In contrast, gallstone impaction induces sphincter of Oddi dysfunction with abnormal bile excretion patterns (38). Clinical studies have revealed exaggerated postprandial bile acid concentration fluctuations in gallstone patients, exacerbating DNA damage through mitochondrial apoptosis pathways (46). Gallbladder polyps, often arising in a background of chronic cholecystitis, are linked to the hypersecretion of pro-inflammatory cytokines such as IL-6, which can promote remote neoplastic progression via angiogenic and invasive pathways (47, 48).

This mechanistic heterogeneity explains the observed risk gradient: cholecystectomy exerts prolonged effects through dual-pathway synergy (bile acid dysmetabolism and microbiota disruption), whereas polyps primarily mediate remote effects via inflammatory cytokines. Although both pathologies involve bile acid composition alterations, their spatiotemporal inflammatory patterns (pulsatile vs. sustained) may partially account for risk stratification.

### 4.3 Clinical and demographic effect modifiers

The substantial statistical heterogeneity (I² = 84%) and wide 95% prediction intervals indicate that the cholecystectomy–polyp association is not uniform but is modified by other factors. Our subgroup analyses and meta-regression identify key sources of this heterogeneity from clinical/demographic effect modifiers, methodological diversity across studies, and varying degrees of confounding control. Clinical and demographic modifiers constituted a major source of heterogeneity. The most striking disparity was geographical, with East Asian populations exhibiting nearly double the risk of North Americans (OR = 1.95 vs. 1.16). This disparity may stem from gene-environment interactions, particularly differences in dietary patterns (e.g., high refined carbohydrate intake) (49). The elevated risk in East Asian populations implies that refined carbohydrate intake may exert stronger driving effects on the microbiota–DCA axis than the protective role of dietary fiber does, whereas the heterogeneous risk profile in North Americans with prevalent low-fiber consumption might be modulated by subgroup dietary variations (e.g., high-fiber consumers). Furthermore, demographic factors played a role. The stronger association in older individuals (≥50 years: OR = 1.41) may reflect an age-related decline in mucosal resilience. The elevated risk observed in women, particularly within the first post-operative decade (OR = 2.02; Vinikoor et al.), suggesting a potential modulatory role of sex hormones, possibly through interactions with bile acid signaling pathways, and indicating a time-sensitive window of susceptibility (50). Anatomical site was another modifier, with the left colon showing greater susceptibility (OR = 1.82 vs. right colon OR = 1.11; Yamaji et al.), potentially due to the enhanced toxicity of bile acids in its more acidic environment (51). Paradoxical associations between lesion size and risk (e.g., strong correlation with small but not large adenomas) may reflect stage-specific mechanistic influences in early carcinogenesis (52).

Methodological diversity constituted another major source of heterogeneity. The pooling of diverse study designs (e.g., cohort vs. case-control)—which inherently differ in their susceptibility to recall and selection bias—introduced fundamental variation. Furthermore, the operational diagnostic criteria of both exposure and outcome were inconsistent across studies. For exposure, the diagnostic methods for gallbladder conditions varied widely, from self-report to different imaging modalities (ultrasound, CT, MRI, endoscopic ultrasound) with varying accuracy, especially for polyp detection. This heterogeneity in exposure ascertainment likely contributed to measurement error and between-study variability. Crucially, the extent of confounding control was highly variable; while most studies adjusted for age and sex, fewer accounted for dietary patterns, which our subgroup analysis revealed to be a critical modifier. This spectrum of methodological rigor likely contributed to the disparate risk estimates.

### 4.4 Clinical implications and future directions

Our findings advocate for the integration of gallbladder history into personalized CRC risk assessment. The consistent risk gradient suggests that patients with a history of cholecystectomy, particularly those of East Asian descent, females in the early post-operative years, or individuals over 50, could be candidates for earlier initiation of colonoscopy or shortened surveillance intervals. The presence of gallbladder polyps may also warrant heightened clinical vigilance. Future research should prioritize longitudinal studies with serial biospecimen collection to delineate the temporal dynamics of risk and elucidate the precise roles of specific bile acids and microbial taxa. Mechanistic studies using organoid models are needed to confirm the carcinogenicity of post-cholecystectomy bile. Finally, cost-effectiveness analyses are required to translate these epidemiological findings into formal, updated screening guidelines.

### 4.5 Limitations

Our conclusions must be interpreted within the context of several limitations. First, the inherent nature of observational data precludes causal inference. Despite adjustments for key confounders, residual confounding by unmeasured or imperfectly measured factors (e.g., detailed dietary components, physical activity) remains possible. Crucially, reverse causality cannot be ruled out; underlying metabolic conditions such as insulin resistance or a pro-inflammatory state may predispose individuals to both gallbladder diseases necessitating surgery and the development of colorectal polyps. Second, the geographic concentration of studies, particularly on gallbladder pathology in East Asia, limits the generalizability of our findings to other ethnic and populations with different genetic backgrounds and lifestyle patterns. Third, and importantly, the lack of individual-level data on the time since cholecystectomy prevented a dose-response analysis of duration effects, leaving a key clinical question about the temporal dynamics of risk unresolved. Finally, while our comprehensive search strategy focused on major databases, restricting to English and Chinese publications may have introduced language bias.

## 5 Conclusion

This systematic review and meta-analysis of 27 observational studies found evidence of an association between cholecystectomy and an increased risk of colorectal polyps (OR = 1.39, 95% CI: 1.21–1.59), with adenomas showing stronger associations than unclassified polyps. Gallbladder pathologies—particularly polyps (OR = 1.30) and stones (OR = 1.20)—are also associated with elevated risks, though these estimates are lower than that for cholecystectomy. Key findings include the following:

1. Geographical disparities: East Asian populations presented nearly double the risk of North Americans (OR = 1.95 vs. 1.16), potentially linked to unmeasured dietary or genetic modifiers.
2. Methodological influences: Medium-sized studies and those adjusting for smoking/alcohol/BMI yielded higher estimates, whereas dietary adjustment attenuated the effects.
3. Age stratification: Stronger associations in individuals ≥50 years (OR = 1.41) suggest age-related mucosal vulnerability.

The observed risk gradient (cholecystectomy > polyps > stones) may reflect a corresponding gradient in the degree or persistence of biliary and systemic disturbances, although the exact nature of these disturbances requires clarification. However, residual heterogeneity (I²=84% for cholecystectomy), potential publication bias (trim-and-fill adjusted OR = 1.13) and most importantly, the observational nature of all included studies underscores the need for cautious interpretation. Residual confounding (e.g., by diet or metabolic factors) or reverse causality remain plausible alternative explanations. In summary, this study identifies gallbladder status, and particularly a history of cholecystectomy, as a factor associated with a higher prevalence of colorectal polyps. These findings suggest that gallbladder history could be considered in personalized CRC risk assessment, which might help to identify individuals for whom earlier or more intensive colonoscopic surveillance could be evaluated. Future studies should prioritize standardized exposure classification and longitudinal designs to better understand the nature of this association and clarify any underlying causal pathways.

## Data Availability

The original contributions presented in the study are included in the article/**Supplementary Material**. Further inquiries can be directed to the corresponding author.

## Supplementary material

Supplementary Material 1 Search Strategies.

Supplementary Material 2 Study Selection Criteria.

Supplementary Material 3 Statistical Methods and Subgroup Definitions.

Supplementary Figure 1 Subgroup Analysis of Association Between Cholecystectomy and Colorectal Polyp Risk. **(a)** by gender; **(b)** by sample size; **(c)** by adjustment for basic factors (sex/age); **(d)** by adjustment for healthy factors (alcohol/smoking/BMI).

Supplementary Figure 2 Subgroup Analysis of Association Between Gallbladder Pathology and Colorectal Polyp Risk. **(a)** by poly pathology type; **(b)** by study design; **(c)** by age; **(d)** by study quality.

Supplementary Table 1 Association Between Cholecystectomy and Colorectal Polyps: Univariable Meta-Regression.

Supplementary Table 2 Cumulative meta-analysis of Association Between Cholecystectomy and Colorectal Polyps.

Supplementary Table 3 Characteristics of Studies included in Gallbladder Pathology-Colorectal Polyp Association.

Supplementary Table 4 Association Between Gallbladder Pathology and Colorectal Polyps: Univariable Meta-Regression.

Supplementary Table 5 Leave-One-Out Sensitivity Analysis and Trim and Fill Results.

Supplementary Table 6 GRADE Evidence Profile: Association Between Gallbladder-Related Factors and Colorectal Polyp Risk.

## Glossary

BMI: Body Mass Index
CI: Confidence Interval
CRC: Colorectal Cancer
CRN: Colorectal Neoplasms
CRPs: Colorectal Polyps
DCA: Deoxycholic Acid
EMT: Epithelial-Mesenchymal Transition
FXR: Farnesoid X Receptor
GRADE: Grading of Recommendations, Assessment, Development, and Evaluations
HR: Hazard Ratio
IL-6: Interleukin-6
LCA: Lithocholic Acid
MOOSE: Meta-analysis of Observational Studies in Epidemiology
NOS: Newcastle-Ottawa Scale
OR: Odds Ratio
RR: Risk Ratio
PRISMA: Preferred Reporting Items for Systematic Reviews and Meta-Analyses
PROSPERO: International Prospective Register of Systematic Reviews
ROS/RNS: Reactive Oxygen Species/Reactive Nitrogen Species
SCFA: Short-Chain Fatty Acid
SPs: Serrated Polyps
Wnt: Wingless/Integrated signaling pathway

## References

[1] Bray F Laversanne M Sung H Ferlay J Siegel RL Soerjomataram I . Global cancer statistics 2022: GLOBOCAN estimates of incidence and mortality worldwide for 36 cancers in 185 countries. CA Cancer J Clin. (2024) 74:229–63. doi: 10.3322/caac.21834, PMID: 38572751

[2] Vogelstein B Fearon ER Hamilton SR Kern SE Preisinger AC Leppert M . Genetic alterations during colorectal-tumor development. N Engl J Med. (1988) 319:525–32. doi: 10.1056/nejm198809013190901, PMID: 2841597

[3] Snover DC . Update on the serrated pathway to colorectal carcinoma. Hum Pathol. (2011) 42:1–10. doi: 10.1016/j.humpath.2010.06.002, PMID: 20869746

[4] Rex DK Ahnen DJ Baron JA Batts KP Burke CA Burt RW . Serrated lesions of the colorectum: review and recommendations from an expert panel. Am J Gastroenterol. (2012) 107:1315–29; quiz 4, 30. doi: 10.1038/ajg.2012.161, PMID: 22710576 PMC3629844

[5] Clinton SK Giovannucci EL Hursting SD . The world cancer research fund/American institute for cancer research third expert report on diet, nutrition, physical activity, and cancer: impact and future directions. J Nutr. (2020) 150:663–71. doi: 10.1093/jn/nxz268, PMID: 31758189 PMC7317613

[6] Valle L Vilar E Tavtigian SV Stoffel EM . Genetic predisposition to colorectal cancer: syndromes, genes, classification of genetic variants and implications for precision medicine. J Pathol. (2019) 247:574–88. doi: 10.1002/path.5229, PMID: 30584801 PMC6747691

[7] Feng Q Liang S Jia H Stadlmayr A Tang L Lan Z . Gut microbiome development along the colorectal adenoma–carcinoma sequence. Nat Commun. (2015) 6:6528. doi: 10.1038/ncomms7528, PMID: 25758642

[8] Zhang Y Liu H Li L Ai M Gong Z He Y . Cholecystectomy can increase the risk of colorectal cancer: A meta-analysis of 10 cohort studies. PLoS One. (2017) 12:e0181852. doi: 10.1371/journal.pone.0181852, PMID: 28771518 PMC5542607

[9] Yu L Liu W Yan Y Jiang Y Gao X Ruan S . No association between cholecystectomy and risk of colorectal cancer: a meta-analysis of cohort studies. Int J Colorectal Dis. (2023) 38:179. doi: 10.1007/s00384-023-04463-0, PMID: 37368048

[10] Chiong C Cox MR Eslick GD . Gallstones are associated with colonic adenoma: a meta-analysis. World J Surg. (2012) 36:2202–9. doi: 10.1007/s00268-012-1646-5, PMID: 22562454

[11] Polychronidis G Siddiqi H Ali Ahmed F Papatheodorou S Giovannucci EL Song M . Association of gallstone disease with risk of colorectal cancer: a systematic review and meta-analysis of observational studies. Int J Epidemiol. (2023) 52:1424–34. doi: 10.1093/ije/dyad042, PMID: 37071919

[12] Liu YL Wu JS Yang YC Lu FH Lee CT Lin WJ . Gallbladder stones and gallbladder polyps associated with increased risk of colorectal adenoma in men. J Gastroenterol Hepatol. (2018) 33:800–6. doi: 10.1111/jgh.14006, PMID: 28971517

[13] Ocvirk S O’Keefe SJD . Dietary fat, bile acid metabolism and colorectal cancer. Semin Cancer Biol. (2021) 73:347–55. doi: 10.1016/j.semcancer.2020.10.003, PMID: 33069873

[14] Lee C Lin TH Lin CJ Kuo CF Pai BC Cheng HT . A noninvasive risk stratification tool build using an artificial intelligence approach for colorectal polyps based on annual checkup data. Healthcare (Basel). (2022) 10(1):169. doi: 10.3390/healthcare10010169, PMID: 35052332 PMC8776068

[15] Kahn HS Tatham LM Thun MJ Heath CW Jr . Risk factors for self-reported colon polyps. J Gen Intern Med. (1998) 13:303–10. doi: 10.1046/j.1525-1497.1998.00095.x, PMID: 9613885 PMC1496952

[16] Yamaji Y Okamoto M Yoshida H Kawabe T Wada R Mitsushima T . Cholelithiasis is a risk factor for colorectal adenoma. Am J Gastroenterol. (2008) 103:2847–52. doi: 10.1111/j.1572-0241.2008.02069.x, PMID: 18684172

[17] Guo XJ Dong HB Liang HW Fang YB Yu GP Zhu YM . To analyze the risk factors and clinical characteristics of colorectal adenoma in young pe ople. J Clin Intern Med. (2023) 40:103–7.

[18] Zhang CM Lee SH Xing HY . Clinical study on risk factors of colorectal polyps under the new medical model. China Pract Med. (2021) 16:100–2. doi: 10.14163/j.cnki.11-5547/r.2021.33.036

[19] Chen CX Guo CY Du J Mao YS Miu M Zhu ZW . Effect of cholecystectomy on colorectal adenomatous polyps in the elderly. Chin J Gerontol. (2017) 37:4834–5.

[20] Dong K Wu J Wang J Liu HT Yan J Qiao GE . Risk factors for colorectal advanced adenoma. Modern Digestion Intervention. (2024) 29:60–3. doi: 10.3969/j.issn.1672-2159.2024.01.013

[21] Xu W Zhang JY Zheng ZQ Wang T Piao MY Liu H . Value of combined detection of IFOBT, tumor markers, and inflammatory markers in predicting occurrence of advanced colorectal adenoma. World Chin J Digestol. (2021) 29:347–55. doi: 10.11569/wcjd.v29.i7.347

[22] Sandler RS Martin ZZ Carlton NM Holland KL . Adenomas of the large bowel after cholecystectomy. A case-control study. Dig Dis Sci. (1988) 33:1178–84. doi: 10.1007/bf01535797, PMID: 3409804

[23] Schernhammer ES Leitzmann MF Michaud DS Speizer FE Giovannucci E Colditz GA . Cholecystectomy and the risk for developing colorectal cancer and distal colorectal adenomas. Br J Cancer. (2003) 88:79–83. doi: 10.1038/sj.bjc.6600661, PMID: 12556963 PMC2376770

[24] Neugut AI Johnsen CM Forde KA Treat MR Nims C Murray D . Cholecystectomy and adenomatous polyps of the colon in women. Cancer. (1988) 61:618–21. doi: 10.1002/1097-0142(19880201)61:3<618::AID-CNCR2820610333>3.0.CO;2-5, PMID: 3338028

[25] Siddiqui AA Kedika R Mahgoub A . A previous cholecystectomy increases the risk of developing advanced adenomas of the colon. South Med J. (2009) 102:1111–5. doi: 10.1097/SMJ.0b013e3181b85063, PMID: 19864992

[26] Vinikoor LC Galanko JA Sandler RS . Cholecystectomy and the risk of colorectal adenomas. Digestive Dis Sci. (2008) 53:730–5. doi: 10.1007/s10620-007-9912-3, PMID: 17710546 PMC2647516

[27] Neugut AI Murray TI Garbowski GC Forde KA Treat MR Waye JD . Cholecystectomy as a risk factor for colorectal adenomatous polyps and carcinoma. Cancer. (1991) 68:1644–7. doi: 10.1002/1097-0142(19911001)68:7<1644::AID-CNCR2820680730>3.0.CO;2-K, PMID: 1893365

[28] Wang N Jia XF Wang K Liu CX . Clinicopathological features of colorectal serrated lesions and associated risk factors in patients of different ages. Mod Oncol. (2023) 31:3227–31.

[29] Polychronidis G Wang K Lo C-H Wang L He M Knudsen MD . Gallstone disease and risk of conventional adenomas and serrated polyps: A prospective study. Cancer Epidemiol Biomarkers Prev. (2021) 30:2346–9. doi: 10.1158/1055-9965.Epi-21-0515, PMID: 34620626 PMC8643323

[30] Xu YC Xu AG Luo C Chen XH . Relationship between cholecystectomy and colorectal adenoma. J Guangdong Med Univ. (2011) 29:384–6.

[31] Llamas KJ Torlach LG Ward M Bain C . Cholecystectomy and adenomatous polyps of the large bowel. Gut. (1986) 27:1181–5. doi: 10.1136/gut.27.10.1181, PMID: 3781331 PMC1433862

[32] Mannes AG Weinzierl M Stellaard F . Adenomas of the large intestine after cholecystectomy. Gut. (1984) 25:863–6. doi: 10.1136/gut.25.8.863, PMID: 6745725 PMC1432569

[33] Zhuang JH Lee XB Wang MH Zhang ZY Ji XW Jin X . Correlation between cholecystectomy and colorectal polyps. Chin J Gastroenterol. (2011) 16:482–4.

[34] Wang YL Lei W Kang YJ Jiang F Ren MJ Cui HY . Detection rate and risk factors of colorectal polyps in asymptomatic individuals. J Army Med Univ. (2017) 39:2232–7. doi: 10.16016/j.1000-5404.201707133

[35] Luo HS Zhang XX Huang XD . Correlation between cholecystectomy and colorectal adenoma. Acta Med Univ Sci Technol Huazhong. (2014) 43:213–4.

[36] Brenner H Hoffmeister M Stegmaier C Brenner G Altenhofen L Haug U . Risk of progression of advanced adenomas to colorectal cancer by age and sex: estimates based on 840,149 screening colonoscopies. Gut. (2007) 56:1585–9. doi: 10.1136/gut.2007.122739, PMID: 17591622 PMC2095643

[37] Xu F Yu Z Liu Y Du T Yu L Tian F . A high-fat, high-cholesterol diet promotes intestinal inflammation by exacerbating gut microbiome dysbiosis and bile acid disorders in cholecystectomy. Nutrients. (2023) 15(17):3829. doi: 10.3390/nu15173829, PMID: 37686860 PMC10489946

[38] Bernstein H Bernstein C Payne CM Dvorakova K Garewal H . Bile acids as carcinogens in human gastrointestinal cancers. Mutat Res. (2005) 589:47–65. doi: 10.1016/j.mrrev.2004.08.001, PMID: 15652226

[39] Fuchs CD Trauner M . Role of bile acids and their receptors in gastrointestinal and hepatic pathophysiology. Nat Rev Gastroenterol Hepatol. (2022) 19:432–50. doi: 10.1038/s41575-021-00566-7, PMID: 35165436

[40] Yao Y Li X Xu B Luo L Guo Q Wang X . Cholecystectomy promotes colon carcinogenesis by activating the Wnt signaling pathway by increasing the deoxycholic acid level. Cell Commun Signal. (2022) 20:71. doi: 10.1186/s12964-022-00890-8, PMID: 35614513 PMC9131663

[41] Amaral Raposo M Sousa Oliveira E Dos Santos A Guadagnini D El Mourabit H Housset C . Impact of cholecystectomy on the gut-liver axis and metabolic disorders. Clin Res Hepatol Gastroenterol. (2024) 48:102370. doi: 10.1016/j.clinre.2024.102370, PMID: 38729564

[42] Bai X Duan Z Deng J Zhang Z Fu R Zhu C . Ginsenoside Rh4 inhibits colorectal cancer via the modulation of gut microbiota-mediated bile acid metabolism. J Adv Res. (2025) 72:37–52. doi: 10.1016/j.jare.2024.06.028, PMID: 38969093 PMC12147625

[43] Ye C Wu C Li Y Chen C Li X Zhang J . Traditional medicine Xianglian pill suppresses high-fat diet-related colorectal cancer via inactivating TLR4/MyD88 by remodeling gut microbiota composition and bile acid metabolism. J Ethnopharmacol. (2024) 333:118411. doi: 10.1016/j.jep.2024.118411, PMID: 38824980

[44] Xu H Fang F Wu K Song J Li Y Lu X . Gut microbiota-bile acid crosstalk regulates murine lipid metabolism via the intestinal FXR-FGF19 axis in diet-induced humanized dyslipidemia. Microbiome. (2023) 11:262. doi: 10.1186/s40168-023-01709-5, PMID: 38001551 PMC10675972

[45] Adhikari AA Seegar TCM Ficarro SB McCurry MD Ramachandran D Yao L . Development of a covalent inhibitor of gut bacterial bile salt hydrolases. Nat Chem Biol. (2020) 16:318–26. doi: 10.1038/s41589-020-0467-3, PMID: 32042200 PMC7036035

[46] Portincasa P Moschetta A Petruzzelli M Palasciano G Di Ciaula A Pezzolla A . Gallstone disease: Symptoms and diagnosis of gallbladder stones. Best Pract Res Clin Gastroenterol. (2006) 20:1017–29. doi: 10.1016/j.bpg.2006.05.005, PMID: 17127185

[47] Lai JQ Zhao LL Hong C Zou QM Su JX Li SJ . Baicalein triggers ferroptosis in colorectal cancer cells via blocking the JAK2/STAT3/GPX4 axis. Acta Pharmacol Sin. (2024) 45:1715–26. doi: 10.1038/s41401-024-01258-z, PMID: 38684798 PMC11272787

[48] Wang D Dubois RN . The role of COX-2 in intestinal inflammation and colorectal cancer. Oncogene. (2010) 29:781–8. doi: 10.1038/onc.2009.421, PMID: 19946329 PMC3181054

[49] O’Keefe SJ Li JV Lahti L Ou J Carbonero F Mohammed K . Fat, fibre and cancer risk in African Americans and rural Africans. Nat Commun. (2015) 6:6342. doi: 10.1038/ncomms7342, PMID: 25919227 PMC4415091

[50] Henríquez-Hernández LA Flores-Morales A Santana-Farré R Axelson M Nilsson P Norstedt G . Role of pituitary hormones on 17alpha-ethinylestradiol-induced cholestasis in rat. J Pharmacol Exp Ther. (2007) 320:695–705. doi: 10.1124/jpet.106.113209, PMID: 17108234

[51] Ridlon JM Kang DJ Hylemon PB . Bile salt biotransformations by human intestinal bacteria. J Lipid Res. (2006) 47:241–59. doi: 10.1194/jlr.R500013-JLR200, PMID: 16299351

[52] Liu Y Zhang S Zhou W Hu D Xu H Ji G . Secondary bile acids and tumorigenesis in colorectal cancer. Front Oncol. (2022) 12:813745. doi: 10.3389/fonc.2022.813745, PMID: 35574393 PMC9097900

