# Supplementary material for: Association between cholecystectomy/gallbladder pathology and colorectal polyps: a systematic review and meta-analysis
Source: Front Oncol. 2026 Jan 14;15:1724606. doi: 10.3389/fonc.2025.1724606 (PMC12847004; doi:10.3389/fonc.2025.1724606)
Supplement: Supplementary Material 1 — Search Strategies. [file DataSheet1.docx]

**Supplementary Material 1**

**1: Search Strategies**

**Search Strategy Overview**:
The search strategy was organized around two main topics including Gallbladder diseases and their surgical treatment, such as gallstones, gallbladder polyps, and cholecystectomy. Colorectal polyps, such as subtypes such as adenomatous polyps.

Language Restrictions: Studies published in English or Chinese were included.

**Search Databases**:

- PubMed
- Web of science
- Embase
- Cochrane Library
- CNKI (China National Knowledge Infrastructure)
- Wanfang Database
- SinoMed

2025/01/22

**Search Strategy (PubMed)**:1102

( ( ("Colonic Polyps"[Mesh] OR "Intestinal Polyps"[Mesh] OR "Adenoma"[Mesh] OR "Adenomatous Polyps"[Mesh] ) OR ( (Colorectal AND Polyp*) OR (Rectal AND Polyp*) OR (Intestinal AND Polyp*) OR (Colorectal AND Adenoma*) OR (Serrated AND (Polyp* OR Adenoma*)) OR (Hyperplastic AND Polyp*) OR (Adenomatous AND Polyp*) OR (Mixed AND Adenoma AND Polyp*) OR (Inflammatory AND Polyp*) ) ) AND ( ("Gallbladder Diseases"[Mesh] OR "Cholelithiasis"[Mesh] OR "Cholecystectomy"[Mesh] OR "Cholecystitis"[Mesh] OR "Choledocholithiasis"[Mesh]) OR ( (Gallbladder AND (Disease* OR Disorder*)) OR Cholelithiasis OR Gallstone* OR Cholecystectomy OR (Gallbladder AND Surgery) OR (Gallbladder AND Polyp*) OR Cholecystitis OR Choledocholithiasis ) ) )NOT( "Case Reports"[Publication Type]）

**Search Strategy (Embase)**:2518

('colonic polyp'/exp OR 'intestinal polyp'/exp OR 'adenoma'/exp OR 'adenomatous polyp'/exp OR ((colorectal NEAR/3 polyp*):ti,ab) OR ((rectal NEAR/3 polyp*):ti,ab) OR ((intestinal NEAR/3 polyp*):ti,ab) OR ((colorectal NEAR/3 adenoma*):ti,ab) OR ((serrated NEAR/3 (polyp* OR adenoma*)):ti,ab) OR ((hyperplastic NEAR/3 polyp*):ti,ab) OR ((adenomatous NEAR/3 polyp*):ti,ab) OR ((mixed NEAR/3 adenoma NEAR/3 polyp*):ti,ab) OR ((inflammatory NEAR/3 polyp*):ti,ab)) AND ('gallbladder disease'/exp OR 'cholelithiasis'/exp OR 'cholecystectomy'/exp OR 'cholecystitis'/exp OR 'choledocholithiasis'/exp OR ((gallbladder NEAR/3 (disease* OR disorder*)):ti,ab) OR cholelithiasis:ti,ab OR gallstone*:ti,ab OR cholecystectomy:ti,ab OR ((gallbladder NEAR/3 surgery):ti,ab) OR ((gallbladder NEAR/3 polyp*):ti,ab) OR cholecystitis:ti,ab OR choledocholithiasis:ti,ab) NOT 'case report'/exp

**Search Strategy (Web of science)**:947

TS = ('colorectal polyp' OR 'colonic polyp' OR 'rectal polyp' OR 'intestinal polyp' OR 'colorectal adenoma' OR 'serrated polyp' OR 'hyperplastic polyp' OR 'sessile serrated adenoma' OR 'traditional serrated adenoma' OR 'adenomatous polyp' OR 'mixed adenoma polyp') AND TS=('gallbladder disease' OR 'cholelithiasis' OR 'gallstones' OR 'gallbladder disorder' OR 'cholecystectomy' OR 'gallbladder surgery' OR 'gallbladder polyp' OR 'cholecystitis' OR 'choledocholithiasis' OR 'cholecystography')

**Search Strategy (Cochrane)**:35

#1 (Colorectal Polyps):ti,ab,kw OR (Colonic Polyps):ti,ab,kw OR (Rectal Polyps):ti,ab,kw OR (Colorectal Adenomas):ti,ab,kw OR (Serrated Polyps):ti,ab,kw

#2 (Gallbladder Diseases):ti,ab,kw OR (Cholelithiasis):ti,ab,kw OR (Gallstones):ti,ab,kw OR (Gallbladder Disorders):ti,ab,kw OR (cholecystectomy):ti,ab,kw

#3 MeSH descriptor: [Colonic Polyps] explode all trees

#4 MeSH descriptor: [Adenomatous Polyps] this term only

#5 MeSH descriptor: [Intestinal Polyps] explode all trees

#6 #3 OR #4 OR #5

#7 MeSH descriptor: [Gallbladder Diseases] explode all trees

#8 MeSH descriptor: [Gallstones] explode all trees

#9 MeSH descriptor: [Cholecystectomy] explode all trees

#10 #7 OR #8 OR #9

#11 (#1 OR #6) AND (#2 OR #10)

To ensure transparency in the literature retrieval process, the original Chinese search terms used in Chinese databases (e.g., CNKI, Wanfang, SinoMed) are listed below. The original Chinese characters are retained here to clarify their precise semantic and contextual relevance.

**Search Strategy (CNKI)**:167

("结直肠息肉" OR "结肠息肉" OR "直肠息肉" OR "肠道息肉" OR "结直肠腺瘤" OR "锯齿型息肉" OR "增生性息肉" OR "腺瘤性息肉" OR "混合型腺瘤息肉" OR "炎症性息肉")

AND

("胆囊疾病" OR "胆石症" OR "胆囊切除术" OR "胆囊炎" OR "胆总管结石" OR "胆囊息肉" OR "胆囊疾病*" OR "胆结石" OR "胆囊手术" OR "胆囊切除" OR "胆囊炎" OR "胆总管结石")

NOT

("病例报告" OR "动物实验")

**Search Strategy (WanFang)**:350

("结直肠息肉" OR "结肠息肉" OR "直肠息肉" OR "肠道息肉" OR "结直肠腺瘤" OR "锯齿型息肉" OR "增生性息肉" OR "腺瘤性息肉" OR "混合型腺瘤息肉" OR "炎症性息肉")

AND

("胆囊疾病" OR "胆石症" OR "胆囊切除术" OR "胆囊炎" OR "胆总管结石" OR "胆囊息肉" OR "胆囊疾病*" OR "胆结石" OR "胆囊手术" OR "胆囊切除" OR "胆囊炎" OR "胆总管结石")

NOT

("病例报告" OR "动物实验")

**Search Strategy (SinoMed)**: 76

#1 "结直肠息肉"[常用字段:智能] OR "结肠息肉"[常用字段:智能] OR "直肠息肉"[常用字段:智能] OR "肠道息肉"[常用字段:智能] OR "结直肠腺瘤"[常用字段:智能] OR "锯齿型息肉"[常用字段:智能] OR "增生性息肉"[常用字段:智能] OR "腺瘤性息肉"[常用字段:智能] OR "混合型腺瘤息肉"[常用字段:智能] OR "炎症性息肉"[常用字段:智能]

#2 "胆囊疾病"[主题词:扩展] OR "胆石症"[主题词:扩展] OR "胆囊切除术"[主题词:扩展] OR "胆囊炎"[主题词:扩展] OR "胆总管结石"[主题词:扩展] OR "胆囊息肉"[主题词:扩展] OR "胆结石"[常用字段:智能] OR "胆囊手术"[常用字段:智能] OR "胆囊切除"[常用字段:智能]

#3 #1 AND #2

#4 "病例报告"[文献类型] OR "动物实验"[特征词]

#5 #3 NOT #4
