# Supplementary material for: Association between cholecystectomy/gallbladder pathology and colorectal polyps: a systematic review and meta-analysis
Source: Front Oncol. 2026 Jan 14;15:1724606. doi: 10.3389/fonc.2025.1724606 (PMC12847004; doi:10.3389/fonc.2025.1724606)
Supplement: Supplementary Material 2 — Study Selection Criteria. [file DataSheet2.docx]

**Supplementary Material 2**

Table S2. Study Selection Criteria

| Category | Criteria | Details |
| --- | --- | --- |
| Initial Exclusion | 1. Study Design | Non-observational studies (RCTs, reviews, animal studies, case reports) |
|  | 2. Language | Non-English/Chinese publications |
|  | 3. Duplicates | Retain earliest/most complete version of duplicate publications |
|  | 4. Cohort Overlap | Select studies with most comprehensive methods/longest follow-up/largest sample size |
| Final Inclusion | 1. Design | Observational studies (cohort, case-control, cross-sectional) |
|  | 2. Exposure Definition | Cholelithiasis: Radiological/surgical confirmation Gallbladder polyps: Imaging confirmation Cholecystectomy: Surgical/hospital records |
|  | 3. Outcome | Colorectal polyp incidence (adenomatous, serrated, inflammatory) |
|  | 4. Data Quality | Effect sizes extractable (OR/RR/HR with 95% CI) |
|  | 5. Quality Control | Newcastle-Ottawa Scale score ≥6 |
| Final Exclusion | 1. Outcome | Studies reporting invasive carcinoma |
|  | 2. Data Quality | Insufficient outcome data/unvalidated exposure measures |
|  | 3. Gray Literature | Grey literature and conference abstracts were included in the analysis if they contained complete data, otherwise they were excluded. |
