# Supplementary material for: Association between cholecystectomy/gallbladder pathology and colorectal polyps: a systematic review and meta-analysis
Source: Front Oncol. 2026 Jan 14;15:1724606. doi: 10.3389/fonc.2025.1724606 (PMC12847004; doi:10.3389/fonc.2025.1724606)
Supplement: Supplementary Material 3 — Statistical Methods and Subgroup Definitions. [file DataSheet3.docx]

**Supplementary Material 3**

Table S3.1. Subgroup Analysis Definitions

| Analysis_Type | Subgroup | Categories |
| --- | --- | --- |
| Predefined | Pathology Type | Adenoma, Serrated polyps, Unclassified |
|  | Gender | Male, Female, Mixed |
|  | Gallbladder Status | Gallstones, Gallbladder polyps |
| Exploratory | Sample Size | Small (<500), Medium (500-2000), Large (>2000) |
|  | Study Quality | Low (<6), Moderate (6-7), High (8-9) |
|  | Geography | East Asia, North America, Other |
|  | Design | Cross-sectional, Case-control, Cohort |
|  | Age | <60, ≥60, Unreported |
|  | Adjustment | Basic/Health/Dietary factors (Adjusted vs. Unadjusted) |

Table S3.2. Statistical Methods Specification

| Method | Application | Details |
| --- | --- | --- |
| Sensitivity Analysis | Leave-one-out | Sequential removal of individual studies |
|  | Quality-based | Exclusion of studies with NOS <6 |
|  | Trim-and-Fill | Adjustment for publication bias |
| Bias Assessment | Funnel Plots | Visual asymmetry evaluation |
|  | Egger's Test | Statistical test for small-study effects |
|  | Begg's Test | Rank correlation test |
| Meta-Regression | Covariates | Publication year, age, design, NOS score, adjustment level, etc. |
|  | Variance Explained | R² calculation for each covariate |
