# Supplementary figures and images for: Association between cholecystectomy/gallbladder pathology and colorectal polyps: a systematic review and meta-analysis
Source: Front Oncol. 2026 Jan 14;15:1724606. doi: 10.3389/fonc.2025.1724606 (PMC12847004; doi:10.3389/fonc.2025.1724606)

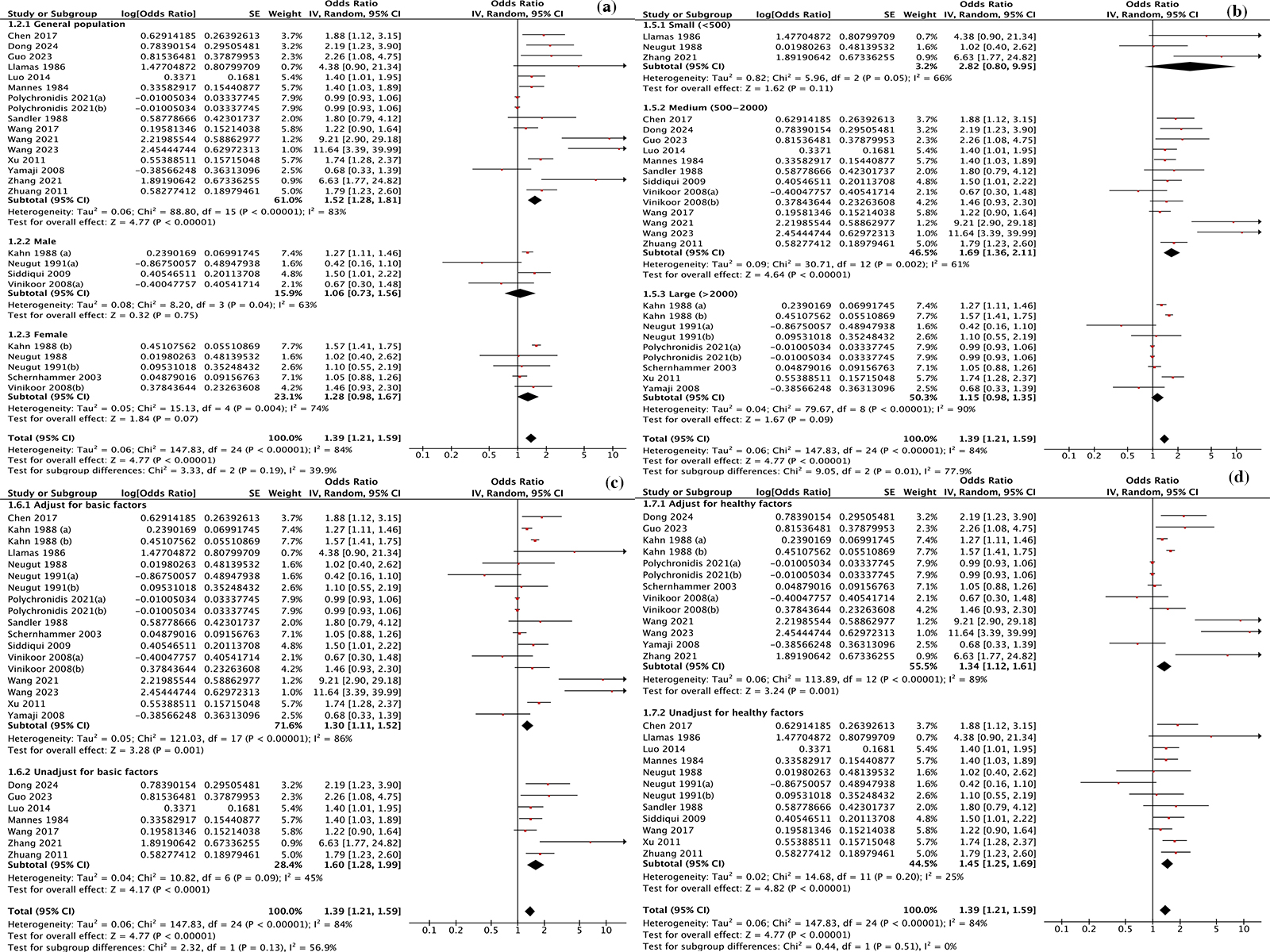

Supplement: Supplementary Figure 1 — Subgroup Analysis of Association Between Cholecystectomy and Colorectal Polyp Risk. (a) by gender; (b) by sample size; (c) by adjustment for basic factors (sex/age); (d) by adjustment for healthy factors (alcohol/smoking/BMI). [file Image1.jpg]

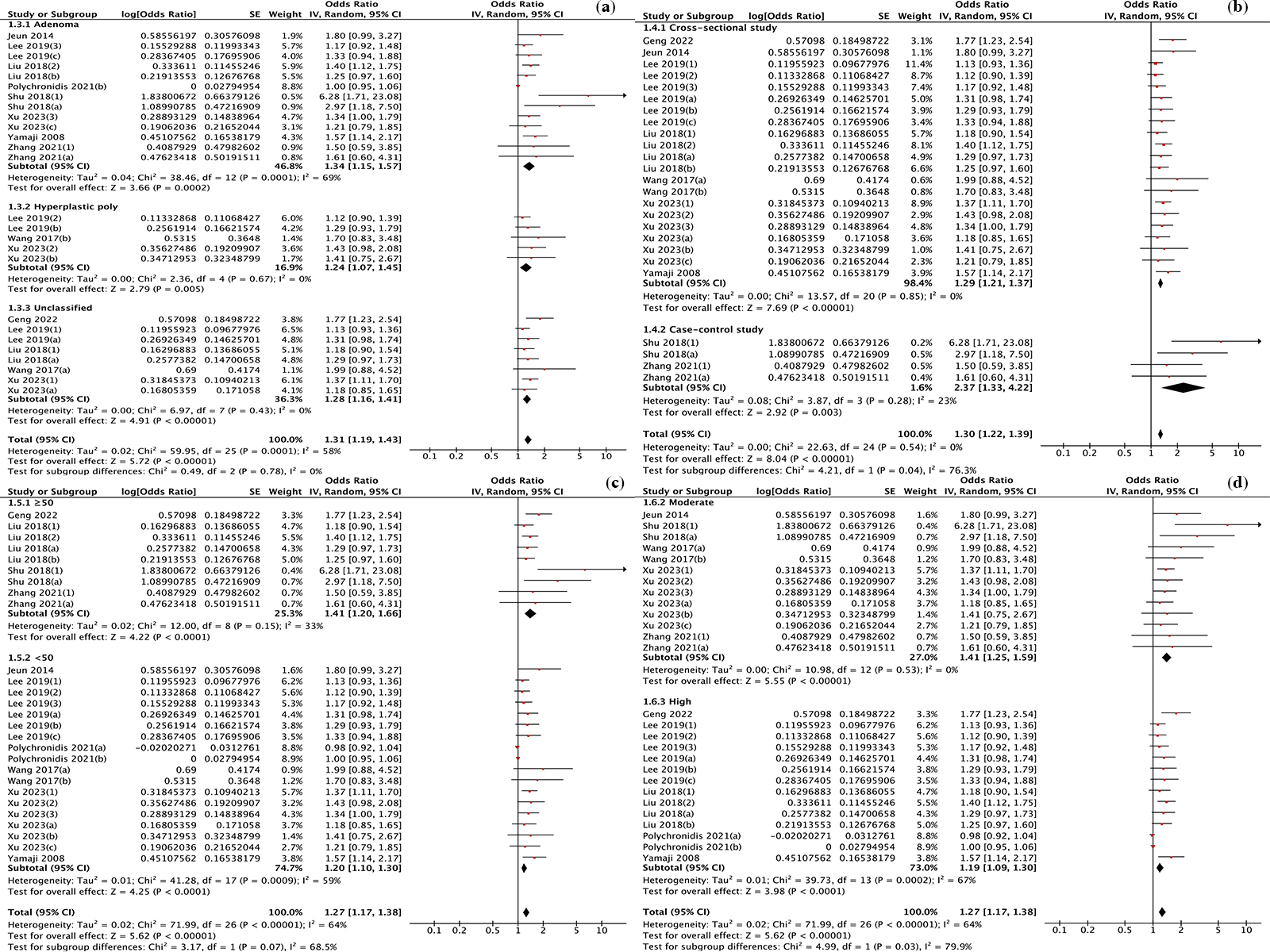

Supplement: Supplementary Figure 2 — Subgroup Analysis of Association Between Gallbladder Pathology and Colorectal Polyp Risk. (a) by poly pathology type; (b) by study design; (c) by age; (d) by study quality. [file Image2.jpg]
