# Supplementary material for: Association between cholecystectomy/gallbladder pathology and colorectal polyps: a systematic review and meta-analysis
Source: Front Oncol. 2026 Jan 14;15:1724606. doi: 10.3389/fonc.2025.1724606 (PMC12847004; doi:10.3389/fonc.2025.1724606)
Supplement: Supplementary Table 1 — Association Between Cholecystectomy and Colorectal Polyps: Univariable Meta-Regression. [file Table1.docx]

***Table S1. Association Between Cholecystectomy and Colorectal Polyps: Univariable Meta-Regression***

| Variable | Subgroup/Unit | Odds Ratio | 95% CI Lower | 95% CI Upper | P value | Tau² | I²(%) | Reference |
| --- | --- | --- | --- | --- | --- | --- | --- | --- |
| PathType | Overall |  |  |  | 0.592 | 0.34 | 93.60 |  |
|  | Unclassified | 1.32 | 0.60 | 2.90 | 0.499 |  |  | Adenoma |
|  | Other | 1.53 | 0.57 | 4.10 | 0.408 |  |  | Adenoma |
| SampleSizeCat | Overall |  |  |  | 0.067 | 0.23 | 95.45 |  |
|  | Medium (500-2000) | 0.70 | 0.27 | 1.80 | 0.464 |  |  | Small (<500) |
|  | Large (>2000) | 0.42 | 0.16 | 1.10 | 0.092 |  |  | Small (<500) |
| NOS_Cat | Overall |  |  |  | 0.366 | 0.32 | 96.53 |  |
|  | Moderate (6-7) | 1.27 | 0.77 | 2.10 | 0.366 |  |  | Low (<6) |
|  | High (8-9) | 1.27 | 0.77 | 2.10 | 0.366 |  |  | Low (<6) |
| PubDecade | Overall |  |  |  | 0.162 | 0.24 | 94.75 |  |
|  | 90s | 0.48 | 0.18 | 1.32 | 0.172 |  |  | 80s |
|  | 00s | 0.70 | 0.36 | 1.38 | 0.319 |  |  | 80s |
|  | 10s | 1.05 | 0.54 | 2.02 | 0.897 |  |  | 80s |
|  | 20s | 1.47 | 0.76 | 2.83 | 0.261 |  |  | 80s |
| Continent | Overall |  |  |  | 0.059 | 0.22 | 95.27 |  |
|  | North America | 0.56 | 0.35 | 0.88 | **0.020** |  |  | East Asia |
|  | Other | 0.89 | 0.35 | 2.25 | 0.812 |  |  | East Asia |
| StudyType | Overall |  |  |  | 0.719 | 0.35 | 96.97 |  |
|  | case-control | 1.41 | 0.61 | 3.26 | 0.432 |  |  | crossectional |
|  | cohort | 1.24 | 0.54 | 2.83 | 0.614 |  |  | crossectional |
| Gender | Overall |  |  |  | 0.164 | 0.27 | 94.94 |  |
|  | Male | 0.54 | 0.28 | 1.05 | 0.081 |  |  | Mixed |
|  | Female | 0.70 | 0.39 | 1.27 | 0.252 |  |  | Mixed |
| AgeCat | Overall |  |  |  | 0.796 | 0.35 | 97.00 |  |
|  | <60 | 0.74 | 0.25 | 2.18 | 0.594 |  |  | Unreported |
|  | ≥60 | 0.86 | 0.28 | 2.70 | 0.803 |  |  | Unreported |
| Hemisphere | Overall |  |  |  | **0.014** | 0.20 | 94.61 |  |
|  | Western | 0.56 | 0.37 | 0.86 | **0.014** |  |  | Eastern |
| Adj_Basic | Overall |  |  |  | 0.370 | 0.32 | 96.56 |  |
|  | adjusted | 0.77 | 0.44 | 1.35 | 0.370 |  |  | unadjusted |
| Adj_Health | Overall |  |  |  | 0.649 | 0.33 | 96.68 |  |
|  | adjusted | 1.13 | 0.67 | 1.89 | 0.649 |  |  | unadjusted |
| Adj_Diet | Overall |  |  |  | 0.667 | 0.33 | 96.15 |  |
|  | adjusted | 0.88 | 0.49 | 1.57 | 0.667 |  |  | unadjusted |
| GenderRatio | Overall |  |  |  | 0.416 | 0.32 | 96.24 |  |
|  | Per unit | 1.20 | 0.78 | 1.83 | 0.416 |  |  |  |
| **Notes:** Continuous variables are presented as 'Per unit'; Categorical variables are shown as comparisons against the reference group; Tau² represents between-study heterogeneity variance, and I² indicates the percentage of heterogeneity. | | | | | | | | |
