# Supplementary material for: Association between cholecystectomy/gallbladder pathology and colorectal polyps: a systematic review and meta-analysis
Source: Front Oncol. 2026 Jan 14;15:1724606. doi: 10.3389/fonc.2025.1724606 (PMC12847004; doi:10.3389/fonc.2025.1724606)
Supplement: Supplementary Table 2 — Cumulative meta-analysis of Association Between Cholecystectomy and Colorectal Polyps. [file Table2.docx]

***Table S2. Cumulative Meta-Analysis Results***

| Study | OR | CI_lower | CI_upper |
| --- | --- | --- | --- |
| Adding Yamaji 2008 (k=1) | 0.6799745 | 0.3337500489 | 1.385364 |
| Adding Guo 2023 (k=2) | 1.2336795 | 0.0005988728 | 2,541.383035 |
| Adding Zhang 2021 (k=3) | 1.9849703 | 0.1240635198 | 31.758788 |
| Adding Chen 2017 (k=4) | 1.8970757 | 0.4711317301 | 7.638832 |
| Adding Dong 2024 (k=5) | 1.9214571 | 0.7739282527 | 4.770465 |
| Adding Wang 2021 (k=6) | 2.4331640 | 0.9449092403 | 6.265456 |
| Adding Sandler 1988 (k=7) | 2.2967480 | 1.0741676122 | 4.910827 |
| Adding Schernhammer 2003 (female) (k=8) | 2.0260611 | 1.0326548762 | 3.975117 |
| Adding Neugut 1988 (a) (k=9) | 1.8816960 | 1.0286392159 | 3.442198 |
| Adding Siddiqui 2009 (b) (k=10) | 1.8120090 | 1.0772484923 | 3.047929 |
| Adding Vinikoor 2008 (a) (k=11) | 1.6695336 | 1.0040348359 | 2.776141 |
| Adding Vinikoor 2008 (b) (k=12) | 1.6359900 | 1.0430714300 | 2.565944 |
| Adding Kahn 1988 (a) (k=13) | 1.5849145 | 1.0600393211 | 2.369680 |
| Adding Kahn 1988 (b) (k=14) | 1.5731983 | 1.0992000987 | 2.251594 |
| Adding Neugut 1991 (a) (k=15) | 1.4830124 | 1.0099714831 | 2.177612 |
| Adding Neugut 1991 (b) (k=16) | 1.4522315 | 1.0176078949 | 2.072484 |
| Adding Wang 2023 (k=17) | 1.6017812 | 1.0595120471 | 2.421589 |
| Adding Polychronidis 2021 (a) (k=18) | 1.5453334 | 1.0483214817 | 2.277980 |
| Adding Polychronidis 2021 (b) (k=19) | 1.4968989 | 1.0390817048 | 2.156430 |
| Adding Xu 2011 (k=20) | 1.5041052 | 1.0698197259 | 2.114686 |
| Adding Llamas 1986 (k=21) | 1.5435691 | 1.1032078599 | 2.159707 |
| Adding Mannes 1984 (k=22) | 1.5283526 | 1.1154692172 | 2.094062 |
| Adding Zhuang 2011 (a) (k=23) | 1.5346188 | 1.1407990512 | 2.064391 |
| Adding Wang 2017 (k=24) | 1.5113884 | 1.1419502230 | 2.000345 |
| Adding Luo 2014 (k=25) | 1.5002003 | 1.1515841409 | 1.954352 |
| Pooled estimate | 1.5002003 | 1.1515841409 | 1.954352 |
