# Supplementary material for: Association between cholecystectomy/gallbladder pathology and colorectal polyps: a systematic review and meta-analysis
Source: Front Oncol. 2026 Jan 14;15:1724606. doi: 10.3389/fonc.2025.1724606 (PMC12847004; doi:10.3389/fonc.2025.1724606)
Supplement: Supplementary Table 3 — Characteristics of Studies included in Gallbladder Pathology-Colorectal Polyp Association. [file Table3.docx]

| *Table S3. Characteristics of Studies included in Gallbladder Pathology-Colorectal Polyp Association* | | | | | | | | | |
| --- | --- | --- | --- | --- | --- | --- | --- | --- | --- |
| Study (Year) | **Pathology Type‡** | **Exposure status§** | **Study Design** | **Adjustment*** | **Quality†** | **Region** | **Sample Size** | **Mean Age** | **Recruitment Period** |
| Wang 2017 | HP+U | GPs +GB | Cross-Sectional | Unadjusted | Moderate | China | 702 | 49.1 | 2013-2016 |
| Lee 2019 | HP+U | GPs +GB | Cross-Sectional | D | High | China | 3,136 | 49.3 | 2008-2009 |
| Liu 2018 | A+U | GPs +GB | Cross-Sectional | D+H | High | China | 9,591 | 54.8 | 2001-2009 |
| Yamaji 2008 | A | GPs +GB | Cross-Sectional | D+H | High | Japan | 5,719 | 46.1 | 1991-2003 |
| Xu 2023 | A+HP+U | GPs +GB | Cross-Sectional | D | Moderate | China | 2,811 | 47.9 | 2020-2023 |
| Shu 2018 | A | GPs +GB | Case-Control | D+H | Moderate | China | 408 | 57.5 | 2016-2018 |
| Polychronidis 2021 | A+S | GPs +GB | Cohort | D+H+N | High | USA | 133,721 | 41.5 | 1991-2012 |
| Zhang 2021 | A | GPs +GB | Case-Control | D | Moderate | China | 103 | 55.6 | 2018-2019 |
| Geng 2022 | U | GPs +GB | Cross-Sectional | D+H | High | China | 1,662 | 55.1 | 2015-2020 |
| Jeun 2014 | A | GPs | Cross-Sectional | D | Moderate | Korea | 581 | 47.6 | 2010-2021 |
| * Adjustment models: D = Demographic factors (age, sex) H = Health factors (smoking, alcohol, BMI) N = Nutritional factors | | | | | | | | | |
| † Quality assessment: High quality (Newcastle-Ottawa Scale 8-9) Moderate quality (NOS 6-7) Low quality (NOS <6) | | | | | | | | | |
| ‡ Pathology classification: A = Adenoma HP = Hyperplastic polyp S = Serrated polyp U = Unclassified lesion § Exposure definitions: GB = Gallstones (cholelithiasis) GP = Gallbladder polyp + indicates concurrent conditions | | | | | | | | | |
