# Supplementary material for: Association between cholecystectomy/gallbladder pathology and colorectal polyps: a systematic review and meta-analysis
Source: Front Oncol. 2026 Jan 14;15:1724606. doi: 10.3389/fonc.2025.1724606 (PMC12847004; doi:10.3389/fonc.2025.1724606)
Supplement: Supplementary Table 4 — Association Between Gallbladder Pathology and Colorectal Polyps: Univariable Meta-Regression. [file Table4.docx]

***Table S4. Association Between Gallbladder Pathology and Colorectal Polyps: Univariable Meta-Regression***

| **Covariate** | **β (SE)** | **p-value** | **Tau²** | **R² (%)** |
| --- | --- | --- | --- | --- |
| Publication Year | -0.018 (0.013) | 0.182 | 0.02 | 8.3 |
| Mean Age | 0.027 (0.004)*** | <0.001 | 3.4e-06 | 100.0 |
| Study Design | 0.591 (0.223)* | 0.014 | 2.4e-06 | 100.0 |
| Quality Score (NOS) | -0.186 (0.028)*** | <0.001 | 2.6e-06 | 100.0 |
| Adjustment Level | 0.141 (0.071) | 0.059 | 2.0e-06 | 100.0 |
| Recruitment Duration | -0.014 (0.002)*** | <0.001 | 2.1e-06 | 100.0 |
| Sex Ratio (M/F) | 0.259 (0.038)*** | <0.001 | 1.8e-06 | 100.0 |
| Country | -0.260 (0.037)*** | <0.001 | 7.8e-06 | 100.0 |
| Time Dimension | -0.854 (0.222)*** | <0.001 | 2.4e-06 | 100.0 |
| Significance levels: *** p<0.001; ** p<0.01; * p<0.05 Tau²: between-study variance; R²: proportion of variance explained | | | | |
