# Supplementary material for: Association between cholecystectomy/gallbladder pathology and colorectal polyps: a systematic review and meta-analysis
Source: Front Oncol. 2026 Jan 14;15:1724606. doi: 10.3389/fonc.2025.1724606 (PMC12847004; doi:10.3389/fonc.2025.1724606)
Supplement: Supplementary Table 5 — Leave-One-Out Sensitivity Analysis and Trim and Fill Results. [file Table5.docx]

Table S5. Leave-One-Out Sensitivity Analysis Results

|  | Pooled Effect (Random Model) | | |
| --- | --- | --- | --- |
| Excluded Study | OR | 95% CI Lower | 95% CI Upper |
| Omitting Wang 2017 (a) | 1.25 | 1.16 | 1.36 |
| Omitting Wang 2017 (b) | 1.26 | 1.16 | 1.36 |
| Omitting Lee 2019(a) | 1.26 | 1.16 | 1.37 |
| Omitting Lee 2019(b) | 1.26 | 1.16 | 1.37 |
| Omitting Lee 2019(c) | 1.26 | 1.16 | 1.37 |
| Omitting Liu 2018 (a) | 1.26 | 1.16 | 1.37 |
| Omitting Liu 2018 (b) | 1.26 | 1.16 | 1.38 |
| Omitting Yamaji 2008 | 1.25 | 1.15 | 1.35 |
| Omitting Xu 2023(a) | 1.27 | 1.16 | 1.38 |
| Omitting Xu 2023(b) | 1.26 | 1.16 | 1.37 |
| Omitting Xu 2023(c) | 1.26 | 1.16 | 1.37 |
| Omitting Shu 2018(a) | 1.25 | 1.16 | 1.35 |
| Omitting Polychronidis 2021(a) | 1.28 | 1.19 | 1.39 |
| Omitting Polychronidis 2021(b) | 1.28 | 1.18 | 1.39 |
| Omitting Zhang 2021 (a) | 1.26 | 1.16 | 1.37 |
| Omitting Geng 2022 | 1.24 | 1.15 | 1.34 |
| Omitting Lee 2019(1) | 1.27 | 1.17 | 1.39 |
| Omitting Lee 2019(2) | 1.27 | 1.17 | 1.38 |
| Omitting Lee 2019(3) | 1.27 | 1.17 | 1.38 |
| Omitting Liu 2018 (1) | 1.27 | 1.16 | 1.38 |
| Omitting Liu 2018 (2) | 1.25 | 1.15 | 1.36 |
| Omitting Jeun 2014 | 1.25 | 1.15 | 1.36 |
| Omitting Xu 2023(1) | 1.25 | 1.15 | 1.37 |
| Omitting Xu 2023(2) | 1.26 | 1.15 | 1.36 |
| Omitting Xu 2023(3) | 1.26 | 1.16 | 1.37 |
| Omitting Shu 2018(1) | 1.25 | 1.16 | 1.34 |
| Omitting Zhang 2021 (1) | 1.26 | 1.16 | 1.37 |
| Pooled estimate | 1.26 | 1.16 | 1.37 |

***Table S5. Trim and Fill Results***

| Parameter | Estimate |
| --- | --- |
| Imputed Studies | 14 |
| Original OR | 1.27 (1.17-1.38) |
| Adjusted OR | 1.09 (0.96-1.23) |
| I² Change | 0.6% → 0.7% |
