# Supplementary material for: Association between cholecystectomy/gallbladder pathology and colorectal polyps: a systematic review and meta-analysis
Source: Front Oncol. 2026 Jan 14;15:1724606. doi: 10.3389/fonc.2025.1724606 (PMC12847004; doi:10.3389/fonc.2025.1724606)
Supplement: Supplementary Table 6 — GRADE Evidence Profile: Association Between Gallbladder-Related Factors and Colorectal Polyp Risk. [file Table6.docx]

| **Table S6. GRADE Evidence Profile: Association Between Gallbladder-Related Factors and Colorectal Polyp Risk** | | | | | | | | | | | |
| --- | --- | --- | --- | --- | --- | --- | --- | --- | --- | --- | --- |
| **Outcome** | **Study Design** | **Risk of Bias** | **Inconsistency** | **Indirectness** | **Imprecision** | **Publication Bias** | **Effect Size** | **Dose-Response** | | **Residual Confounding** | **Overall Quality** |
| **1. Cholecystectomy → Overall colorectal polyp risk** | Observational (cohort/case-control) | **↓ Serious** • 40% of studies lacked adjustment for dietary factors (e.g., red meat intake). • Selection bias in case-control studies (hospital-based controls). | **↓ Serious** • High heterogeneity (I²=84%). • Subgroup differences: East Asia (OR=1.95) vs. North America (OR=1.16). | **No** • All studies targeted adults undergoing endoscopic screening, aligning with the research question. | **No** • Narrow CI (1.21–1.59), excluding null effect. | **↓ Likely** • Trim-and-fill analysis suggested missing studies (adjusted OR=1.13). | **Small effect** • OR=1.39 (moderate but consistent). | **No** • Insufficient data on time since cholecystectomy. | **↑ Possible** • Unmeasured confounders (e.g., bile acid levels) may underestimate true effect. | | **LOW** |
| **2. Gallbladder polyps → Adenomatous polyp risk** | Observational (cross-sectional/cohort) | **↓ Serious** • Gallbladder polyps diagnosed via ultrasound only (no histologic confirmation). • 30% of studies did not adjust for smoking. | **↓ Serious** • Moderate heterogeneity (I²=59%). • Conflicting results in Western vs. Asian studies. | **No** • Polyps histologically confirmed across studies. | **No** • CI=1.17–1.38, excluding null. | **↓ Likely** • Funnel plot asymmetry (Egger’s test P=0.03). | **Small effect** • OR=1.30. | **No** • Limited data on polyp size/number. | **No** • No evidence of residual confounding. | | **VERY LOW** |
| **3. Gallstones → Serrated polyp risk** | Observational (cohort) | **↓ Serious** • Gallstone size inconsistently reported. • 50% of studies used self-reported gallstone history. | **No** • Low heterogeneity (I²=30%). | **No** • Outcome defined by histology (serrated polyps). | **No** • CI=1.08–1.32. | **No** • Symmetric funnel plot (Begg’s test P=0.22). | **Small effect** • OR=1.20. | **↑ Present** • Trend between gallstone diameter and risk (P=0.06). | **↑ Possible** • Residual confounding by BMI. | | **LOW** |
